# Advancements in CRISPR-based therapies for ocular pathologies: from disease mechanisms to intervention strategies

**DOI:** 10.7150/thno.117085

**Published:** 2026-01-01

**Authors:** Qinghua Li, Qingdong Bao, Sihan Zhao, Fan Wu, Yang Li, Kaiyuan Wang, Wenlong Li, Hua Gao

**Affiliations:** 1 State Key Laboratory Cultivation Base, Shandong Provincial Key Laboratory of Ophthalmology, Eye Institute of Shandong First Medical University, Qingdao 266071, China.; 2 Eye Hospital of Shandong First Medical University (Shandong Eye Hospital), Jinan 250021, China.; 3 School of Ophthalmology, Shandong First Medical University, Jinan 250001, China.; 4 State Key Laboratory of Structural Chemistry, Fujian Institute of Research on the Structure of Matter, Chinese Academy of Sciences, Fuzhou 350002, China.; 5 Department of Pharmaceutics, Wuya College of Innovation, Shenyang Pharmaceutical University, Shenyang 110016, China.; 6 Departments of Diagnostic Radiology, Surgery, Chemical and Biomolecular Engineering, and Biomedical Engineering, Yong Loo Lin School of Medicine and College of Design and Engineering, National University of Singapore, Singapore 119074, Singapore.

**Keywords:** CRISPR gene therapy, eye diseases, ocular immune privilege, genetic modification, clinical application

## Abstract

Eye diseases caused by genetic mutations affect over 2.2 billion people worldwide. The development of CRISPR technology has opened exciting possibilities for how we diagnose and treat these conditions. However, designing effective CRISPR systems, managing potential risks, and considering the ethical questions around gene therapy in clinical practice are major challenges. To move forward successfully, it's important to evaluate how practical CRISPR-based treatments are for eye diseases from a clinical perspective, while also understanding how CRISPR systems work. In this review, we start by covering the basic principles behind CRISPR technology and explore its different types. Next, we look at various ways CRISPR is being used in eye research and treatments, from early studies to new clinical approaches. Lastly, we address the regulatory environment and ethical issues involved, discussing existing rules, safety concerns, and guidelines for genetic modifications in medical settings. Our goal is to share new insights into innovative treatments for eye diseases and to support the safe use of CRISPR in clinical eye care. This review aims to be a helpful resource for researchers, doctors, and regulators working on CRISPR-based therapies.

## Introduction

The human eye is one of the body's most detailed organs, tasked with transmitting visual information to the brain. An estimated about 27% of people worldwide suffer from various eye conditions, with around 2.2 billion individuals experiencing some forms of visual impairment or blindness 1. Sight-threatening ocular diseases primarily include hereditary diseases such as corneal dystrophy, aniridia, Leber congenital amaurosis (LCA), and retinitis pigmentosa (RP), and multifactorial diseases like corneal scarring, glaucoma, and age-related macular degeneration (AMD) 2. With in-depth investigation into disease mechanisms, an increasing number of specific gene mutations associated with ocular diseases have been identified, serving either as genetic risk factors or pathogenic variants. These diseases have long lacked effective curative treatments, while gene therapy designed to directly repair or compensate for defective genes holds promise for achieving a one-time cure 3. The eye's unique immune privilege makes it an ideal target for gene therapy. The clinical evidence for this privileged status is primarily reflected in the high success rate of corneal transplantation and the feasibility of experimental tissue grafting into the eye, indicating a relatively tolerant ocular immune system toward foreign vectors 4. Anatomically, the corneal and blood-retinal barrier (BRB) confine delivery vectors within the eye, allowing local administration of gene therapy medicines to limit widespread side effects 5. Plus, the transparent nature of the eye makes it easier to monitor treatment progress directly, which is highly beneficial for clinical applications 6.

Gene therapy has achieved revolutionary breakthroughs in the field of ophthalmology 7, 8. For instance, gene augmentation therapy represented by Luxturna delivers functional copies of the RPE65 gene to retinal cells *via* subretinal injection, restoring the visual cycle and improving visual function in patients 9. However, current gene replacement therapy is limited to autosomal recessive genetic disorders. When facing autosomal dominant mutations (such as autosomal dominant RP, aniridia) and multifactorial diseases (AMD, glaucoma), simply supplementing a normal gene copy cannot overcome the toxic effects of the original mutant protein 10. Consequently, gene therapy is rapidly evolving towards more complex and precise approaches.

Thanks to advances in genetic engineering-most notably the discovery of Clustered regularly interspaced short palindromic repeats (CRISPR) and its associated proteins (Cas)-there has been a major shift in how ophthalmic conditions are researched and treated 11, 12. Originally identified as part of bacterial immune defense 13, CRISPR/Cas system has quickly become a groundbreaking tool for genome editing because of its high precision and adaptability 14. CRISPR/Cas9 is the most renowned and widely utilized version of CRISPR tools. Its core components consist of the Cas9 protein and a single guide RNA (sgRNA), which incorporates both the trans-activating CRISPR RNA (tracrRNA) and CRISPR RNA (crRNA) 15. The sgRNA directs the Cas9 protein to bind and cleave the target DNA sequence by recognizing a protospacer adjacent motif (PAM) located downstream of the target genomic site, generating double-strand breaks (DSBs) 16. Then, the cellular intrinsic genome repair mechanisms-non homologous end joining (NHEJ) and homology directed repair (HDR)-are activated to achieve the insertion, deletion, or replacement of the target sequence 16.

**Figure 1:**
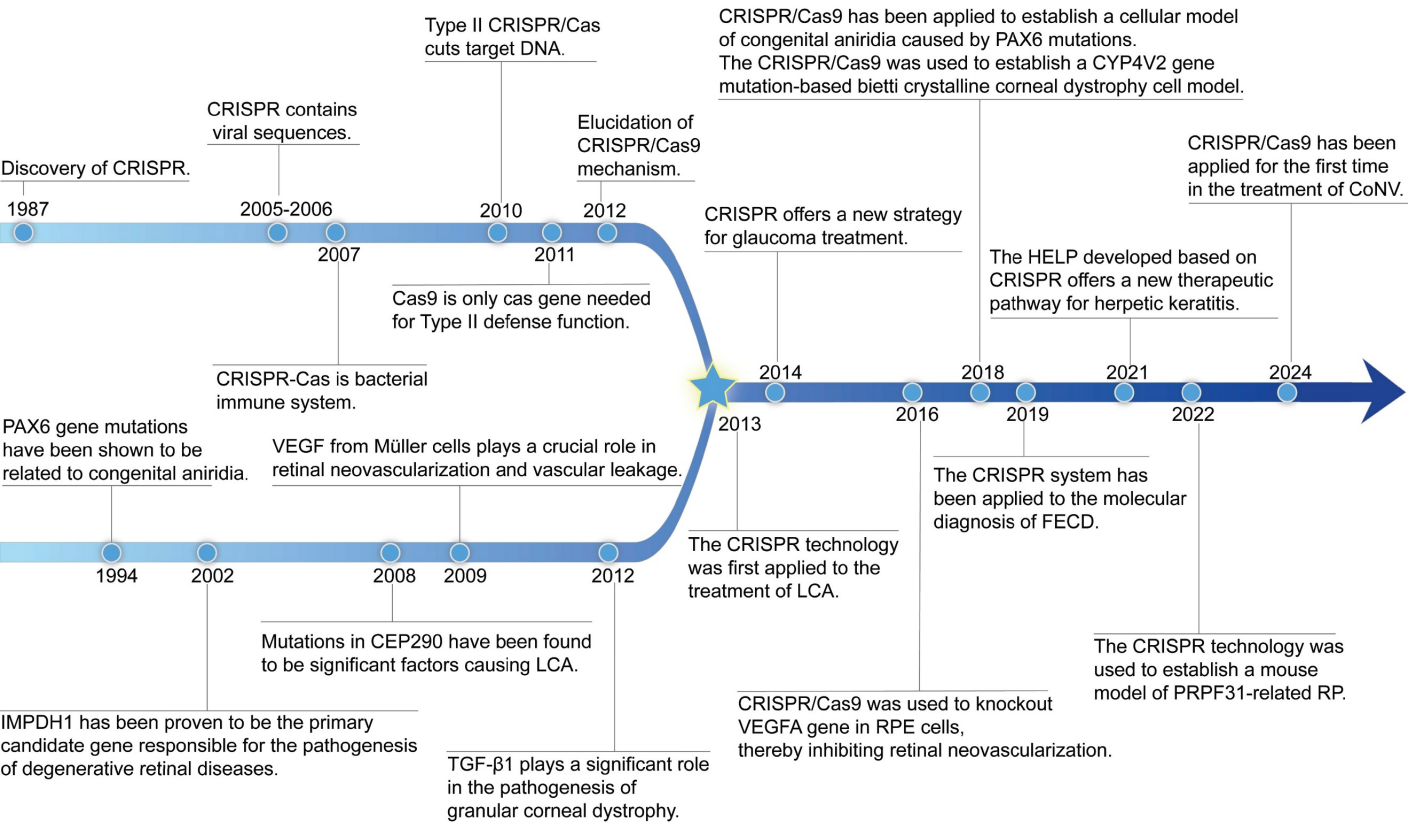
The review of the history of CRISPR system development, the discovery journey of genetic loci for eye diseases, and the advancement of CRISPR treatment in eye diseases.

Since Doudna and Charpentier's pioneering work in 2012 demonstrated CRISPR's effectiveness in mammalian cells, this technology has become essential for revealing disease mechanisms, studying genes linked to eye conditions, and creating experimental models 17, 18** (Figure 1)**. A notable milestone was reached in 2020 when the first *in vivo* application of CRISPR/Cas9 in LCA10 patients was performed, involving subretinal delivery targeting *CEP290* mutations 19. This demonstrated that gene editing directly within the eye can be a feasible therapeutic strategy. Beyond hereditary diseases, CRISPR also shows promise for multifactorial diseases caused by infections, immune responses, or abnormal vessel growth. For instance, using CRISPR to target herpes simplex virus (HSV) DNA in viral keratitis to interrupt viral replication and lower recurrence risks 20. CRISPR gene editing technology overcomes the limitation of conventional gene replacement therapy, which only target autosomal recessive disorders, and significantly expanding the scope of gene therapy applications 21. Compared to gene augmentation therapies relying on transgenic expression, CRISPR/Cas achieves precise *in situ* edit of target genes, eliminating potential risks of non-physiological expression associated with exogenous therapeutic genes 22.

Despite promising results seen in early studies and laboratory experiments, there are still several major challenges to overcome before CRISPR can be widely used in clinics 23, 24. Issues such as unintended effects on other parts of the genome, challenges in delivering the editing tools effectively, improving how well the editing works, and ensuring safety over the long term are all critical concerns. This tension between the exciting potential to treat diseases and the need to address safety and ethical concerns emphasizes the necessity for careful oversight and thorough scientific evaluation when applying CRISPR in eye health. Therefore, conducting a detailed and honest review of how CRISPR technology might be used to treat eye diseases is essential to move forward safely and responsibly.

In this review, we take a close look at the latest developments, the healing possibilities, and the challenges involved in using gene editing with CRISPR in eye medicine** (Table 1)**. We also discuss how current rules and future pathways could shape the field. This review is intended as a helpful resource for researchers, ophthalmologists, and regulators involved in advancing and applying CRISPR-based treatments in ophthalmology. By offering this thorough overview, we hope to provide fresh perspectives on treating eye diseases and support the safe development of CRISPR technology in clinical practice.

## Classification and Mechanisms of CRISPR Gene Editing Systems

The CRISPR system, first identified in *Escherichia coli*, is a highly advanced immune mechanism found in bacteria and other prokaryotes 25. It helps defend against invading genetic materials, such as viruses called bacteriophages or foreign DNA segments 26. This powerful defense involves two main parts, each with its own structure and function 27. The first component consists of characteristic DNA elements featuring short palindromic repeat sequences of 20-40 base pairs (bp), namely CRISPR. The second part includes the Cas genes and the proteins they produce.

**Figure 2:**
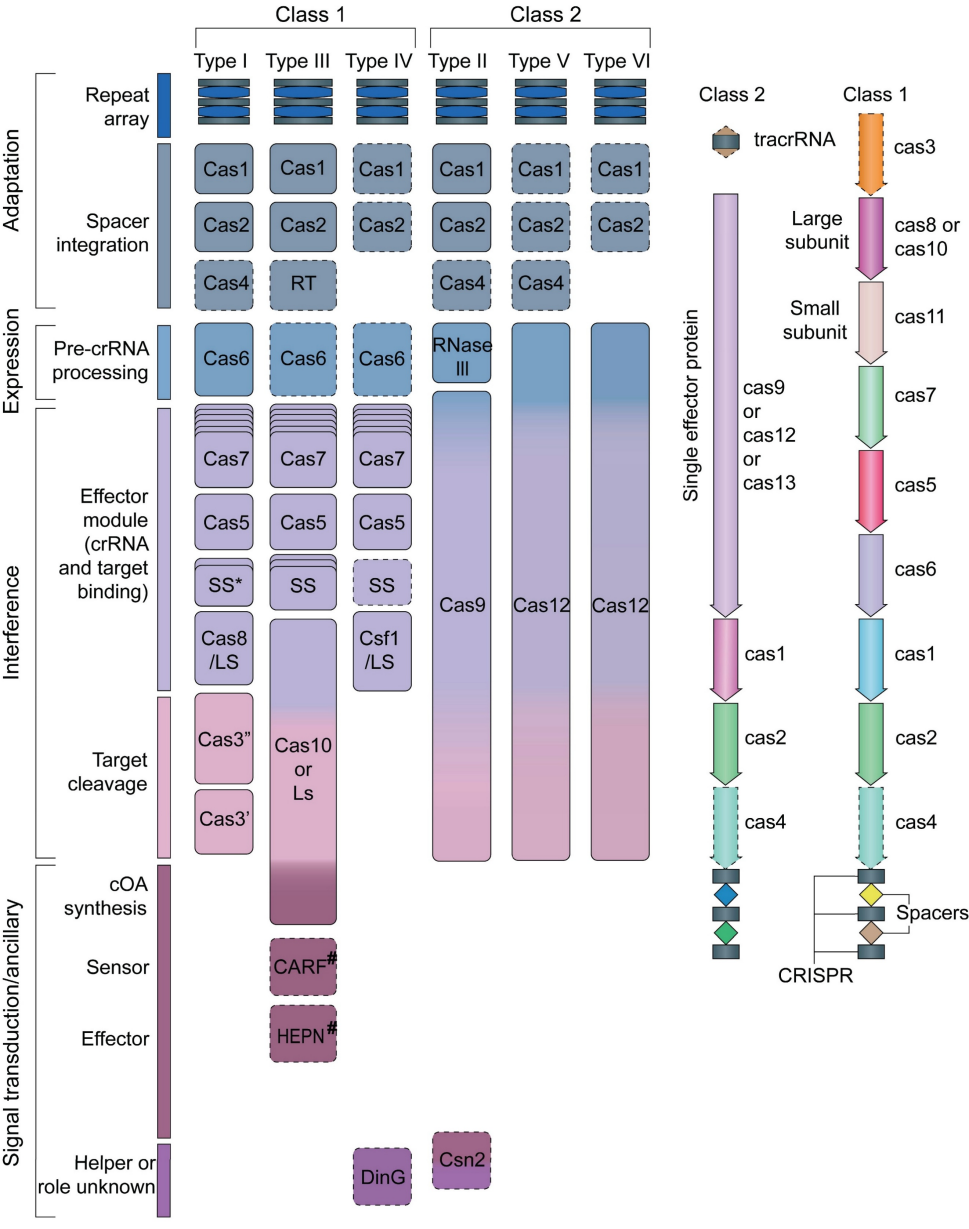
Schematic diagram of the classification of CRISPR/Cas gene editing systems. Adapted with permission from 28, copyright 2019, the authors.

Modern classification divides CRISPR systems into two main types, with six subtypes **(Figure 2)** 28. Understanding these differences helps scientists grasp the wide range of functions and uses of CRISPR-Cas systems. This detailed molecular knowledge has changed how we manipulate genes and develop new treatments. Today, CRISPR-Cas tools are central to modern biology and medicine, opening up exciting possibilities for research and therapy.

### Class 1 CRISPR-Cas Systems

Class 1 CRISPR-Cas systems (comprising Types I, III, and IV) are characterized by their unique multisubunit effector complexes, which enable precise gene editing through careful interactions between proteins and nucleic acids 29. However, Class 1 CRISPR-Cas systems, due to their reliance on multi-subunit complexes composed of multiple Cas proteins, have long been considered to face numerous challenges in practical applications:

(1). Inefficient assembly of multi-subunit complexes causes unstable editing efficiency. Type I systems require Cas1-Cas2 mediated spacer acquisition, and the Cascade complex participates in the interference stage 30. This complexity makes system simplification and application more challenging.

(2). The effector complexes of Class 1 systems typically consist of 4-5 protein subunits, with a total gene size generally exceeding 3.8 kb (e.g., Type I-E reaches 4.4 kb), limiting their application in *in vivo* gene therapy 31.

(3). Class 1 systems exhibit higher uncontrollability. For instance, Type III-E systems consume ATP and accumulate toxic ITP, which may lead to cellular metabolic disturbances 32.

Due to the above-mentioned reasons, no related literature on the application of Class 1 CRISPR-Cas systems in ophthalmology has been reported to date.

### Class 2 CRISPR-Cas Systems

Compared with Class 1 systems, Class 2 systems have significantly greater advantages in ocular pathologies research due to their structural simplicity, operational convenience, and extensive applicability 33. Class 2 systems only require a single effector protein (e.g., Cas9, Cas12a, Cas13a) to bind with gRNA to function, offering simpler operation and higher editing efficiency 34. Then, Class 2 effector proteins have a compact size, enabling compatibility with virus vectors for *in vivo* delivery 35. Additionally, the simple structure of Class 2 systems facilitates engineering modifications to enhance safety 36.

Class 2 systems include three main types-Type II, Type V, and Type VI-each with its own unique way of working and special features.

#### The Type II CRISPR-Cas System

**Figure 3:**
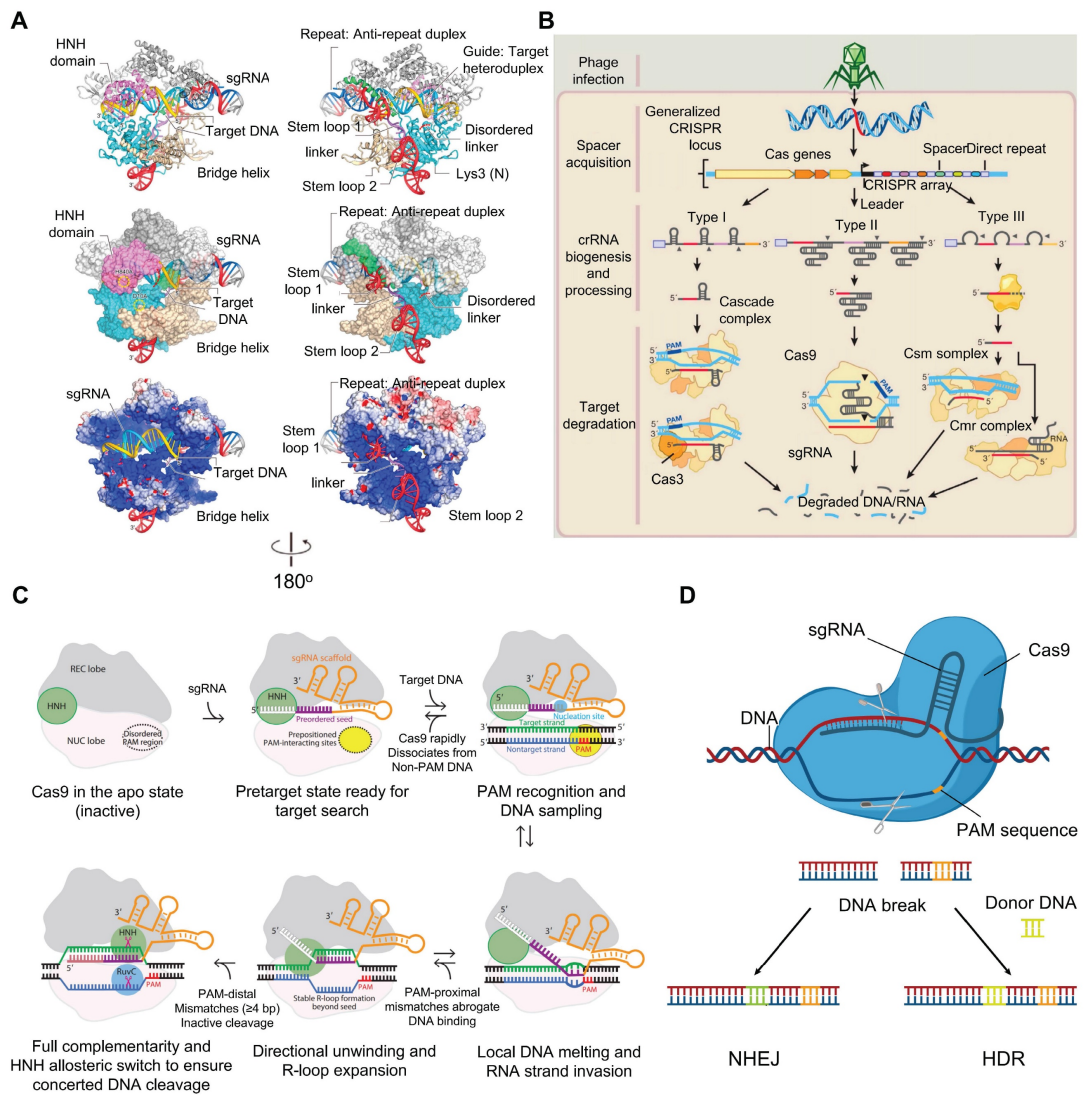
** The mechanism of action and related structures of CRISPR/Cas9: (A)** The overall structure of the Cas9-sgRNA-DNA three-dimensional system. Adapted with permission from 41, copyright 2014, Elsevier Inc. **(B)** DNA interference in CRISPR-Cas9-mediated bacterial adaptive immunity. Adapted with permission from 16, copyright 2014, Elsevier Inc. **(C)** Schematic diagram of the mechanism of CRISPR/Cas9-mediated target DNA recognition and cleavage. Adapted with permission from 39, copyright 2017 by Annual Reviews.** (D)** The general mechanism of CRISPR/Cas9 genome editing.

The Type II CRISPR-Cas system, often called CRISPR/Cas9, is the most well-known and widely used version of the CRISPR-Cas tools 37 **(Figure 3A)**. As a groundbreaking gene editing technology, CRISPR/Cas9 originates from the natural immune mechanism of prokaryotic organisms **(Figure 3B)**. It mainly involves the Cas9 protein, along with a gRNA integrating tracrRNA with crRNA 15. Cas9 acts as the main protein responsible for cutting DNA 38. The crRNA, which is produced from CRISPR sequences, guides Cas9 to the specific DNA target, while tracrRNA binds to crRNA's repeat regions and to Cas9 to help in maturing the RNA and enabling DNA cutting 39. The PAM sequence is a short DNA motif located just downstream of the target DNA sequence, plays an essential role in helping Cas9 recognize its specific target 40. When the gRNA pairs with the target DNA, Cas9 creates an R-loop structure that activates its two main nuclease domains: HNH and RuvC 41. The HNH domain cuts the DNA strand complementary to the guide, while RuvC cuts the opposite strand, resulting in a double-strand break (DSBs). The cell then repairs this break either through nonhomologous end joining (NHEJ) or homology directed repair (HDR) 42 **(Figure 3C-D)**.

The NHEJ repair is currently the primary pathway for gene knockout and has been widely applied in both hereditary ophthalmic diseases and multifactorial diseases 44. The NHEJ repair pathway directly ligates broken DNA, bypassing the time-consuming template search step required in homologous recombination. This process introduces insertions or deletions (indels) that frequently cause frameshift mutations, thereby deactivating genes with dominant-negative gain-of-function mutations 45. For instance, CRISPR/Cas9 exploits NHEJ pathway to disrupt the glaucoma-associated *MYOC* gene and lower intraocular pressure 46. Unlike NHEJ, HDR requires the provision of an exogenous homologous repair template to guide the cell for precise repair 47. However, HDR mainly occurs during the cell division phase (S/G2 phase), and its editing efficiency is usually below 20%, which greatly limits its application in *in vivo* gene editing 47.

Homology-Independent Targeted Integration (HITI) is another breakthrough CRISPR/Cas9-mediated gene editing technology that overcomes the cell cycle dependency of traditional HDR, allowing efficient and precise gene knock in in both dividing and non-dividing cells (such as retinal cells) 48. Its core principle is to utilize the NHEJ pathway to achieve targeted integration of exogenous DNA. After Cas9 cleaves both the target gene and the donor DNA, the cellular NHEJ machinery integrates the donor DNA into the target locus during DSBs repair 48. HITI, leveraging its cell cycle independent high efficiency integration capability to directly supplement the corresponding wild-type gene fragment, may become a transformative tool for ophthalmic gene therapy, particularly for inherited retinal diseases (IRDs) 49. Utilizing HITI technology to repair the CYP4V2 gene mutation in a humanized CYP4V2 mutant mouse model has achieved precise DNA repair and functional protein expression, offering potential for the treatment of Bietti crystalline dystrophy 50.

Moreover, the CRISPR/Cas9 system has also given rise to several gene editing tools:

(1). The Adenine base editor (ABE) is made by fusing nuclease-deficient Cas proteins such as nickase Cas9 (nCas9) with adenine deaminase 51. Under the guidance of sgRNA, nCas9 binds to and nicks a single strand of the target DNA. Adenine deaminase then converts the target adenine (A) to inosine (I), which pairs like guanine (G), achieving an A-to-G conversion 52. Like ABE in structure and editing, cytosine base editor (CBE) is formed by fusing nCas9 with cytosine deaminase 51. It converts target cytosine (C) to uracil (U), which is later changed to thymine (T), thus achieving a C-to-T conversion. Base Editor precisely edits adenine bases at specific genomic sites without causing DSBs and with high specificity and low off-target effects 53.

(2). Prime editor (PE) consists of a reverse transcriptase fused to a Cas9 nickase and a prime editing guide RNA (pegRNA) containing both a gRNA and a template RNA 54. pegRNA guides the prime editing protein to the target site. The Cas9 nickase then creates a single strand break on the target DNA strand. Subsequently, the reverse transcriptase initiates reverse transcription using the pegRNA as a template and directly polymerizes the DNA product onto the nicked target DNA strand 55. PE overcomes the limitations of traditional CRISPR/Cas9 and base editors, enabling targeted insertions, deletions, and all 12 types of base substitutions without the need for DSBs or donor DNA templates 56. Due to these advantages, PE has shown great application potential in the field of ophthalmology, especially in the treatment of IRDs. A recently developed PE system effectively corrected the *PDE6B* Y347X mutation associated with RP, successfully restored PDE6B protein expression, and significantly improved retinal function 57.

(3). CRISPR/dCas9 is a gene editing tool derived from the CRISPR/Cas9 system 58. The dCas9 protein is engineered through mutagenesis of key amino acid residues in Cas9, resulting in loss of DNA cleavage activity while retaining DNA-binding capability 59. By fusing dCas9 to transcriptional activators (e.g., VPR, p65, RTA) or repressors (e.g., KRAB domain), CRISPR/dCas9 enables targeted activation or suppression of gene expression 60. After dCas9 binds to the gene promoter or enhancers, activators can recruit the transcription machinery, to promote gene transcription (CRISPR activation, CRISPRa); whereas repressors sterically hinder RNA polymerase binding/elongation, suppressing gene transcription (CRISPR interference, CRISPRi) 61. For instance, dCas9 fused to the VPR showed effective transcriptional activation and long-term expression of cone photoreceptor-specific M-opsin in rhodopsin-deficient mouse models of RP 62. Diverging from DSB-dependent CRISPR/Cas9 editing, the CRISPR/dCas9 platform introduces no permanent genetic modifications and allows reversible toggling of gene expression states while preserving genomic integrity. Collectively, CRISPR/dCas9 provides a safer and more precise approach to transcriptional regulation.

The Type II CRISPR-Cas system has become a widely used tool for gene editing, regulation, and research. Its simplicity, efficiency, and accuracy make it an essential resource for studying and treating eye diseases and developing new gene therapies.

#### The Type V CRISPR-Cas System

The Type V CRISPR-Cas system relies on proteins from the Cas12 family as its main effectors 63. These proteins have a distinctive two-part (bilobed) structure: one part, called the REC domain, is responsible for recognizing the crRNA and target DNA, while the other part, known as the NUC domain, contains RuvC nuclease activity that cuts DNA 64-66. This system is mainly divided into subtypes V-A, which includes Cas12a, and V-B, which includes Cas12b. Cas12a can process its own crRNA, identify specific AT-rich sequences called PAMs, and make staggered cuts in DNA using its RuvC domain 65. This differs from Cas9, which recognizes NGG PAM sequences and cuts to produce blunt ends. Newer subtypes like V-M (Cas12m2, for example) don't cut double stranded DNA but can still turn off gene expression by strongly binding to DNA helping defend against mobile genetic elements like viruses. The unique structures and functions of Cas12 proteins open up exciting possibilities for gene editing and regulation 67. Because of these features, Cas12 proteins are becoming increasingly important tools in gene editing research today.

#### The Type VI CRISPR-Cas System

The type VI CRISPR-Cas system uses Cas13 protein as the core effector protein and is known for its ability to use its HEPN domain for ribonuclease activity to target RNA 68, 69. The subtypes are further divided into four classes VI-A to VI-D, with the most extensively studied being VI-A, represented by Cas13a. Upon crRNA binding, this activates the HEPN domain of Cas13 traditionally *via* structural conformations, which allows for sequence-specific recognition of foreign RNA that is independent of PAM sequences 70. It has two modes: cis cleavage, in which it accurately degrades target RNA, and trans-cleavage, an indiscriminate collateral effect where it breaks down adjacent RNA 71. Such collateral activity has been harnessed for molecular diagnostics, especially through SHERLOCK technology that reports high-sensitivity detection of pathogens including Zika virus and COVID-19 *via* fluorescent signal amplification 72, 73. The system mutually partakes in microbial immunity and biomedicine as functionalities through the unitary mechanism of combinatorial nanoparticle-mediated virus identification, trigger-based defensing and multi-modal response execution into a precise antiviral defense and transportable diagnostic utility.

The contents of the above discussion represent the current classifications of the CRISPR gene editing systems. As science continues its path of exploration, we expect that new types of CRISPR systems will be uncovered. Herein, we will explore the use of the CRISPR gene editing system for ocular diseases.

## Molecular Mechanisms and Potential CRISPR-Based Therapies

### Anterior Eye Disease

**Figure 4:**
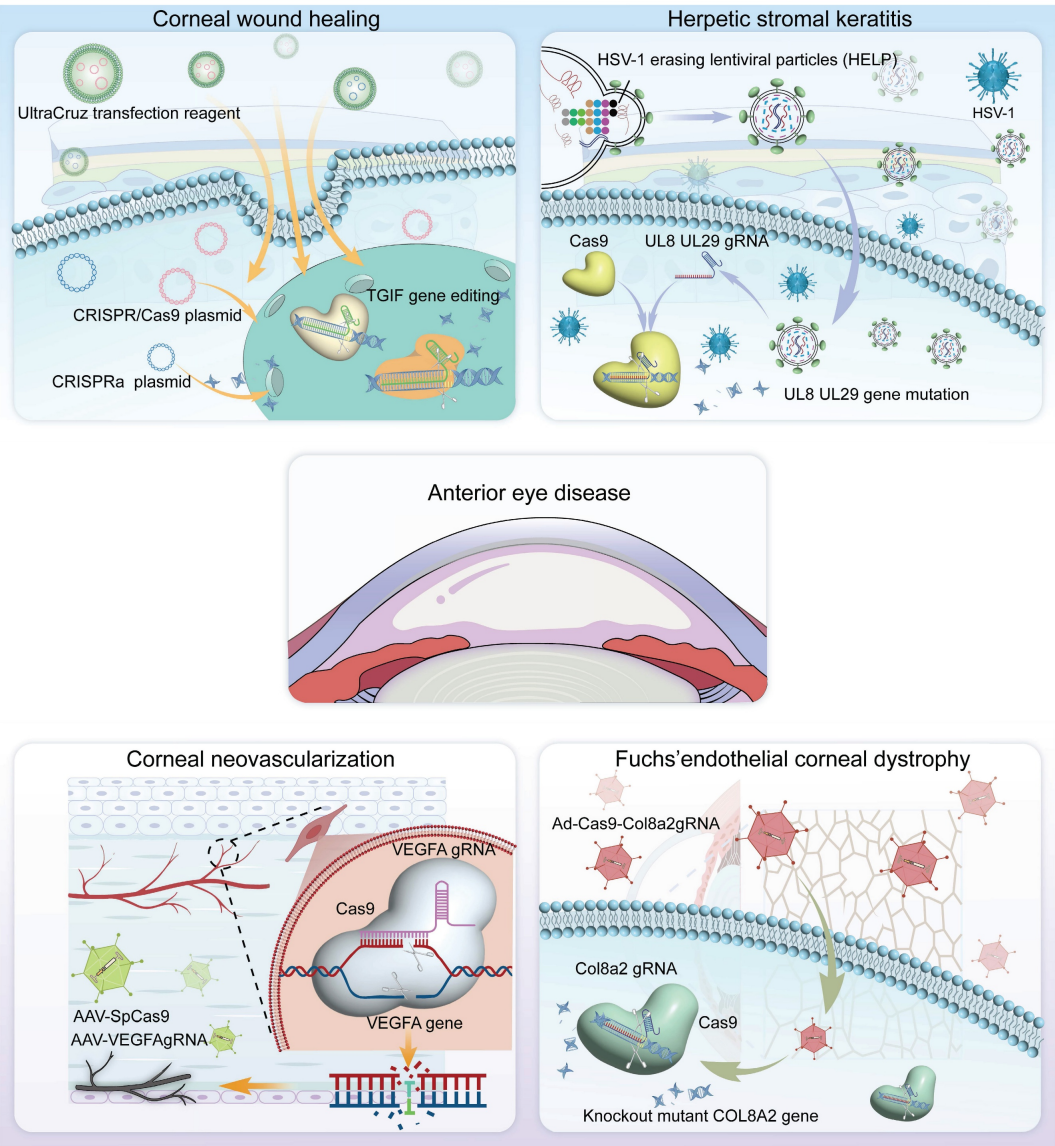
Representative applications of CRISPR gene editing system in anterior segment diseases.

CRISPR technology has immense potential in the treatment of ocular diseases, with a growing focus on its use in corneal-related conditions. In this chapter, we summarize the pathogenesis of these conditions and explore the feasibility of using novel CRISPR-based gene therapies for corneal disease treatment** (Figure 4)**.

#### Corneal Scar and Corneal Wound Healing

**Figure 5:**
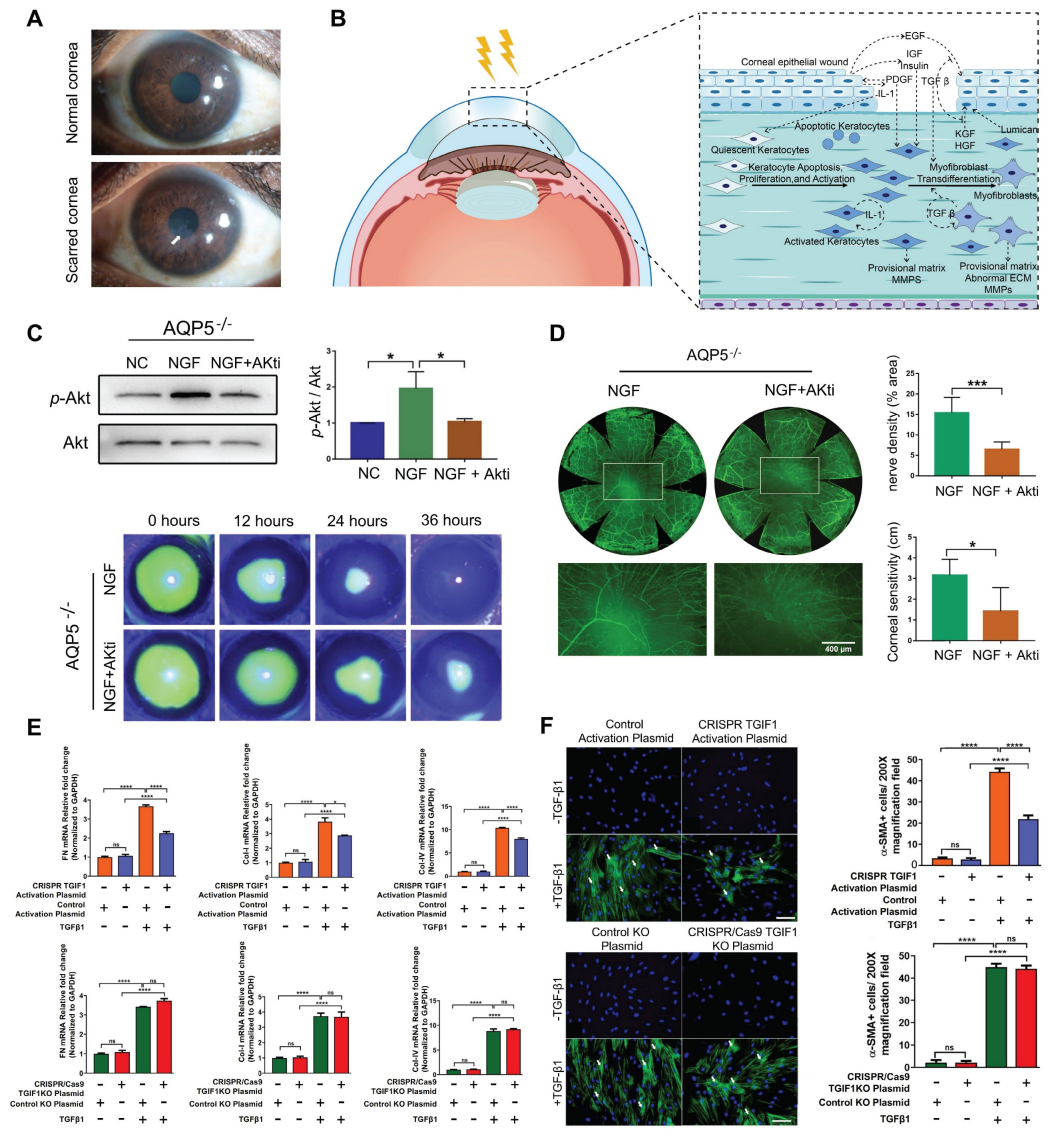
** Presentation of key data from recent studies on corneal scar and wound healing: (A)** Images of corneal scar. Adapted with permission from 74, copyright 2022, the authors. **(B)** Molecular mechanisms involved in corneal repair, remodeling, and regeneration after injury. **(C-D)** Injecting NGF in *AQP5* knockout mice promotes corneal epithelial wound healing and nerve regeneration *via* Akt pathway activation. Adapted with permission from 76, copyright 2021, Elsevier Inc. **(E)** Effects of CRISPR/Cas9 mediated *TGIF1* activation or knockout on the expression of pro-fibrotic genes. Adapted with permission from 77, copyright 2022, Elsevier Ltd. **(F)** CRISPR-activated *TGIF1* strongly inhibited α-SMA protein expression. Adapted with permission from 77, copyright 2022, Elsevier Ltd.

Corneal scarring is a critical condition that compromises corneal transparency, and severe corneal injuries have been recognized as one of the major factors leading to corneal fibrosis and scar formation 74** (Figure 5A-B)**. Currently, corneal transplantation is the only option for treating corneal scar. As a result, several studies aim to restore corneal transparency by manipulating the gene expression of certain genes. Utilizing the CRISPR/dCas9 activation system to enhance the expression of Sex Determining Region Y-Box 2 (SOX2) successfully increased the proliferative capacity, and quantity of human corneal endothelial cells (hCECs), thereby holding promise for addressing the challenge of hCECs regeneration 75. Liu *et al.* used CRISPR/Cas9 technology to generate an *AQP5* gene knockout mouse model to study corneal injury. AQP5 deficiency reduced nerve growth factor (NGF) expression in the corneal epithelium, thereby inhibiting the activation of the Akt signaling pathway and adversely affecting corneal epithelial wound healing and nerve regeneration. Akt inhibitor (Akti) was used to block the reactivation of the Akt signaling pathway, and when applied alongside NGF, it reversed the corneal regeneration effects of NGF in CRISPR/Cas9 mediated *AQP5*-KO mice 76** (Figure 5C-D)**.

Recent research in this field has increasingly focused on the TGF-β signaling pathway. CRISPR/dCas9-mediated CRISPRa enables targeted gene expression activation without genomic sequence alteration, serving as a powerful tool for functional genomics research. Tripathi *et al.* built *in vitro* models by knocking out (CRISPR/Cas9) or activating (CRISPRa) the *TGIF1* gene using CRISPR technology and identified that activation of *TGIF1* gene significantly prevented the human corneal stromal fibroblasts (hCSFs) from changing into corneal myofibroblasts (CMFs) and inhibited profibrotic gene expression 77** (Figure 5E-F)**. The study showed that activation of *TGIF1* inhibited corneal fibrosis and promoted tissue repair, suggesting a potential gene therapy strategy for corneal scars.

#### Herpetic Keratitis

**Figure 6:**
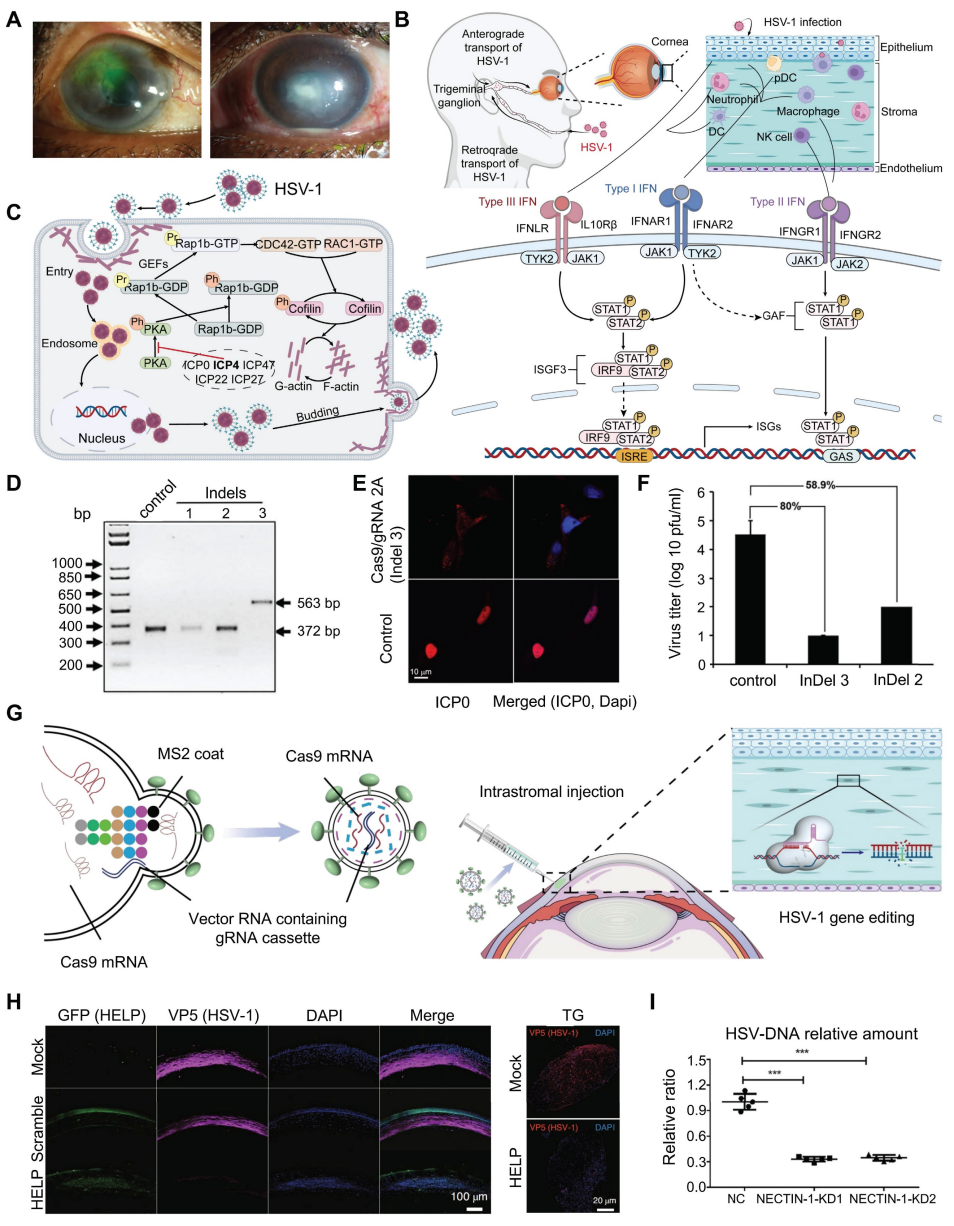
** Presentation of research results related to the treatment of herpes keratitis based on the CRISPR system: (A)** Slit-lamp photos reveal central corneal stromal opacity in a patient with HSK. Adapted with permission from 89, copyright 2024, the authors. **(B)** HSV-1 infection process. Adapted with permission from 90, copyright 2023, the authors. Licensee MDPI, Basel, Switzerland. **(C)** *ICP4* activates the Rap1b-CDC42-RAC1-Cofilin pathway, promoting HSV-1 invasion. Adapted with permission from 84, copyright 2023, the authors. Licensee MDPI, Basel, Switzerland.** (D)** Nucleic acid gel electrophoresis analysis of three types of InDel mutations. Adapted with permission from 85, copyright 2016, the authors. **(E)** Representative confocal images of L7 cells after immunostaining with an antibody against *ICP0*. Adapted with permission from 85, copyright 2016, the authors.** (F)** Viral production assay. Adapted with permission from 85, copyright 2016, the authors. **(G)** The construction of the HELP system. Adapted with permission from 20, copyright 2021, the authors.** (H)** HELP blocks HSV-1 infection of corneas and trigeminal ganglion. Adapted with permission from 20, copyright 2021, the authors.** (I)** *NECTIN-1* knockout reduces HSV-DNA expression. Adapted with permission from 88. copyright 2022, the authors.

Herpetic keratitis (HK), especially the herpetic stromal keratitis (HSK) type, is very destructive and can result in corneal blindness 78** (Figure 6A)**. HSK is driven primarily by immune mediated inflammation from interactions of HSV infected resident corneal cells and infiltrating inflammatory cells 79-81** (Figure 6B)**. *ICP0* and* ICP4* are two important genes that are needed for the replication and reactivation of HSV-1 82-84** (Figure 6C)**. Roehm *et al*. delivered Cas9 and sgRNA targeting the *ICP0* gene into *ICP0*-complementing L7 cells *via* plasmid transfection, which successfully introduced InDel mutations at the target site and significantly reduced HSV-1 viral yield 85 **(Figure 6D-F)**. Chen and colleagues employed an AAV1-SaCas9 system to target and edit the *ICP4* gene of HSV-1 within trigeminal ganglion neurons, significantly suppressing viral replication and establishing a foundation for developing curative therapies against HSK 86.

Moreover, the CRISPR/Cas9 system has been utilized to regulate other important HSV genes. Yin *et al.* used a CRISPR/Cas9-based HELP (HSV-1 erasing lentiviral particles) system by incorporating spCas9 mRNA and gRNAs that interrupt the essential HSV-1 genes *UL8* and *UL29* into mRNA-carrying lentiviral particles, demonstrating potent HSV-1 replication inhibition 20** (Figure 6G)**. HELP system cleared HSV-1 not only in the cornea but also retrogradely transported to the trigeminal ganglion where latent HSV-1 virus was cleared** (Figure 6H)**. Utilizing Virus-like particles (VLP) delivery technology, BDgene Therapeutics has developed BD111, a CRISPR/Cas9 based gene editing drug that directly targets and cleaves the HSV-1 genome, thereby enabling precise treatment for herpetic keratitis. In investigator-initiated trials (IITs), BD111 has demonstrated favorable safety and tolerability (ClinicalTrials.gov Identifier: NCT0456079). Additionally, construction of HSV-1 mutants with *UL7* gene alterations *via* the CRISPR/Cas9 system demonstrated that the UL7 protein is essential for viral infection and proliferation, in addition to elucidating at the molecular level its mechanism of regulating viral replication and pathogenicity *via* the transcription of α4 genes 87. These insights provided an important theoretical basis for the development of novel anti-HSV-1 therapeutics.

In addition to interfering with the replication of HSV, blocking HSV receptors and their adhesion processes represents a viable therapeutic strategy for herpes simplex keratitis. Using the CRISPR/Cas9 system, sgRNAs targeting the second exon of the nectin cell adhesion molecule 1 (*NECTIN-1*) gene were designed by Hong's group, and the delivered Cas9 and sgRNAs to hCECs *via* a lentiviral vector resulting in significant downregulating the expression of NECTIN-1, which significantly reduces HSV-1 infection and improves cell survival 88** (Figure 6I)**.

#### Corneal Dystrophy

**Figure 7:**
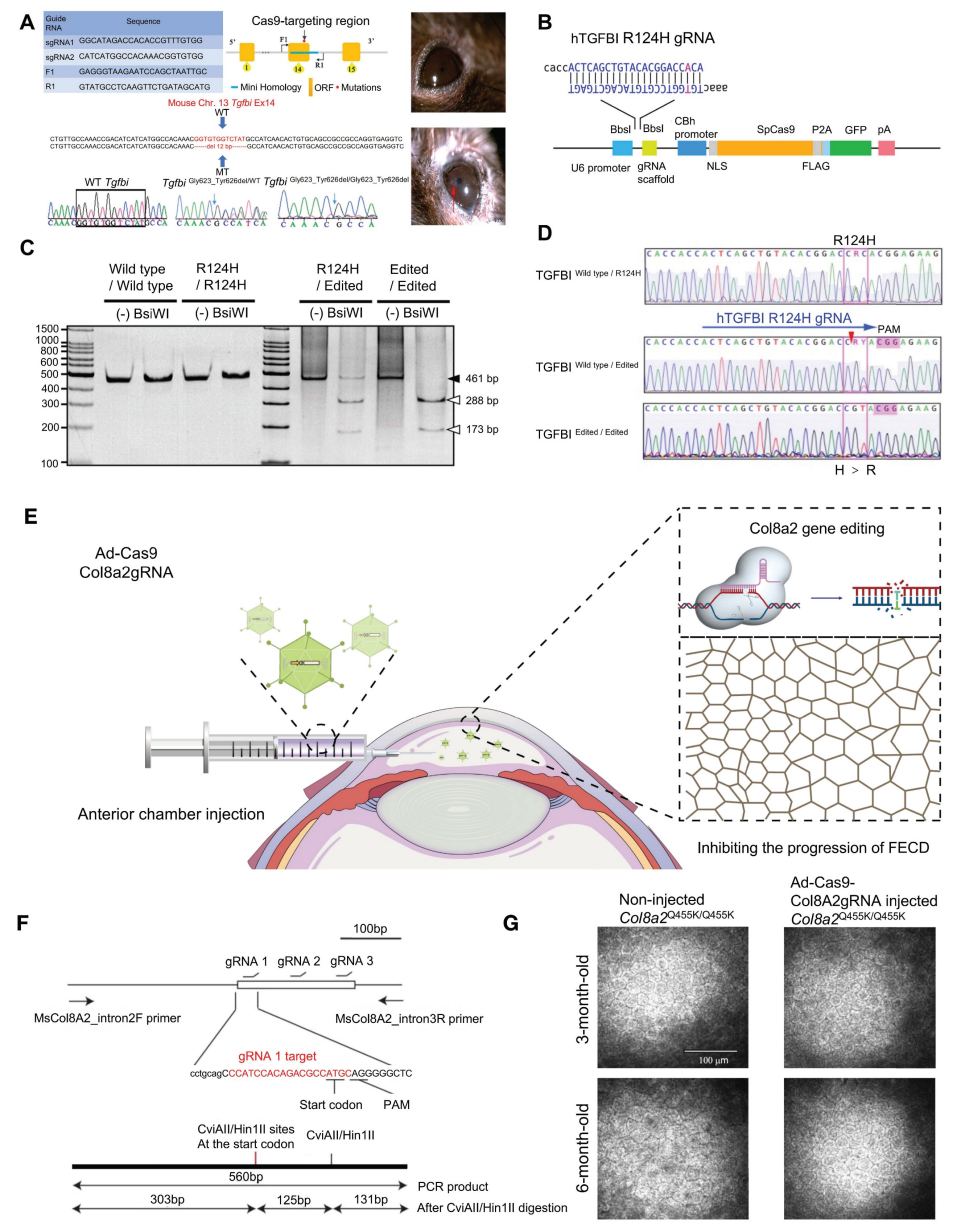
**Recent key findings in the study of corneal dystrophy: (A)** TGFBI-related murine TBCD model was made by CRISPR/Cas9. Adapted with permission from 93, copyright 2021, the authors. **(B)** Linear structure of the plasmid for CRISPR/Cas9-mediated HDR of a *TgfbI* mutation. Adapted with permission from 94, copyright 2017, the authors. **(C-D)** Correction of the mutation in *TgfbI* mutant keratocytes using CRISPR-mediated HDR. Adapted with permission from 94, copyright 2017, the authors.** (E)** Construction and mechanism of Ad-Cas9-Col8a2 gRNA. **(F)** Schematic diagram of Col8a2 gRNA design and PCR product restriction endonuclease detection. Adapted with permission from 96, copyright 2021, Uehara *et al.* **(G)** Ad-Cas9-Col8a2 gRNA significantly reduced corneal endothelial cell loss. Adapted with permission from 96, copyright 2021, Uehara *et al.*

Corneal dystrophy is a hereditary progressive disease involving all layers of the cornea, with complex pathogenesis and structural pathological changes 91. Corneal dystrophies are mainly categorized into four major types: epithelial and subepithelial dystrophies, epithelial-stromal TGF-β1-induced dystrophies, stromal dystrophies, and endothelial dystrophies 92. In a study of TGF-β1-induced corneal dystrophy, CRISPR/Cas9 technology was utilized to establish a humanized mouse model of TGF-β1-linked Thiel-Behnke corneal dystrophy (TBCD) reproduced the corneal opacity phenotype observed in human patients 93** (Figure 7A)**. Taketani's team successfully achieved *in vitro* gene correction of the R124H mutation in the *TgfbI* gene within primary human corneal keratocytes derived from a granular corneal dystrophy patient, utilizing CRISPR/Cas9 technology combined with a single-stranded oligodeoxynucleotide donor template 94. Fuchs' endothelial corneal dystrophy (FECD) is the best-characterized of the endothelial dystrophies 95** (Figure 7B-D)**. Mutations in the *Col8a2* gene are known causes of FECD, resulting in alterations to the α2 chain of type VIII collagen encoded by this gene. *Col8a2* mutant knock-in mice exhibited corneal endothelial excrescences known as guttae, as well as the endothelial cell loss and disruption of the hexagonal structure, which are hallmarks of human FECD. The research of Uehara indicated that *in vivo* adenovirus-mediated delivery of SpCas9 and gRNA allowed efficient ablation of the *Col8a2* mutants in corneal endothelial cells, preventing cell death and restoring normal corneal endothelial function in a mouse model of early-onset FECD 96** (Figure 7E-G)**.

#### Corneal Neovascularization (CoNV)

**Figure 8:**
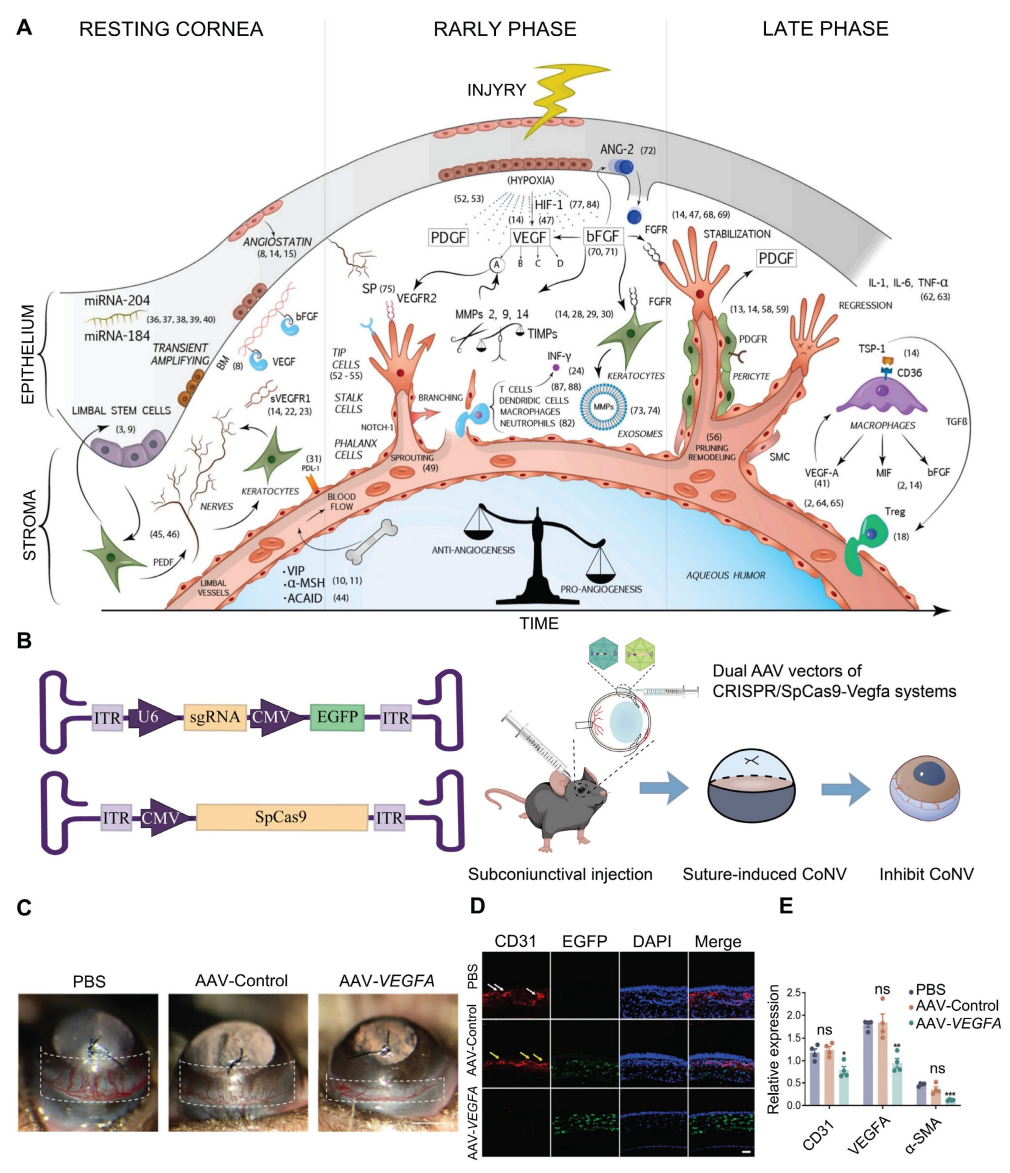
** Presentation of key data from representative studies on CoNV: (A)** Summary Schematic Diagram of the Molecular Pathways in CoNV. Adapted with permission from 100, copyright 2021, Elsevier Ltd. **(B)** CRISPR/SpCas9-*Vegfa* dual AAV vector system. Adapted with permission from 99, copyright 2024, the authors.** (C)** CRISPR/SpCas9-*Vegfa* system reduces suture-induced CoNV. Adapted with permission from 99, copyright 2024, the authors. **(D-E)** CD31, VEGFA, and α-SMA protein expression in corneal tissue. Adapted with permission from 99, copyright 2024, the authors.

The transparency of a healthy cornea is mainly ensured by maintaining a delicate balance of antiangiogenic and proangiogenic factors in its microenvironment 97. When the cornea is injured, such as in herpes simplex keratitis, chemical burns, and corneal transplant rejection, the secretion of angiogenic factors increases, inducing angiogenesis. VEGFA is a crucial angiogenic factor, and its upregulation is a primary contributor to corneal pathologies 98** (Figure 8A)**. Building on this mechanism, Zeng *et al.* designed and constructed a CRISPR/Cas9 system targeting the *Vegfa* gene and delivered it to mouse corneas *via* subconjunctival injection **(Figure 8B)**. This system significantly suppressed suture-induced CoNV in mice and downregulated protein expression levels of VEGFA, CD31 (endothelial cell marker), and α-SMA (myofibroblast/smooth muscle cell marker) 99** (Figure 8C-E)**. The core advantage of the highly precise CRISPR/Cas9 gene editing system lies in its ability to specifically recognize and cleave target gene sequences. In the treatment of CoNV, CRISPR/Cas9 technology offers significant improvement over traditional pharmacological approaches, such as anti-VEGFA antibodies and small-molecule inhibitors, by directly modifying the genome to produce sustained biological effects.

### Midsection Eye Disease

**Figure 9:**
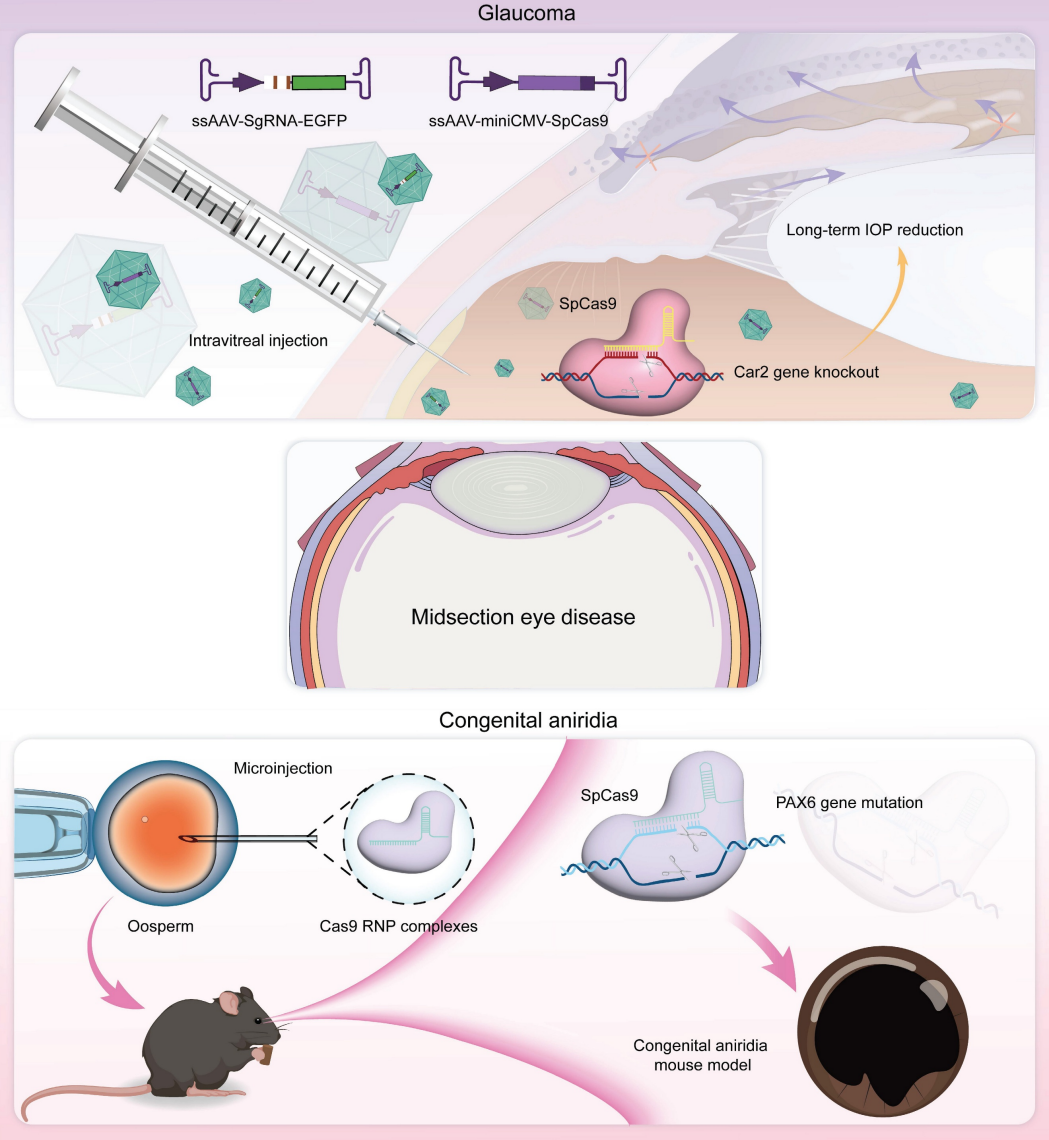
Representative applications of CRISPR system in midsection eye diseases.

The ocular midsegment, which includes the iris, lens, and ciliary body, plays a crucial role in light modulation and the maintenance of visual function. Among the diseases of the ocular midsegment, glaucoma is the most prevalent and impactful, significantly reducing patients' quality of life, whereas traditional therapies often fail to address the underlying issues effectively. Leveraging the precise gene repair capabilities of CRISPR, we anticipate the potential to revolutionize the treatment of these conditions, achieving lasting and effective therapeutic outcomes through genetic intervention. In the following sections, we review the genetic mechanisms underlying these diseases and explore the potential applications of CRISPR technology in ocular midsegment disorders, along with prospects for future development** (Figure 9)**.

#### Glaucoma

**Figure 10:**
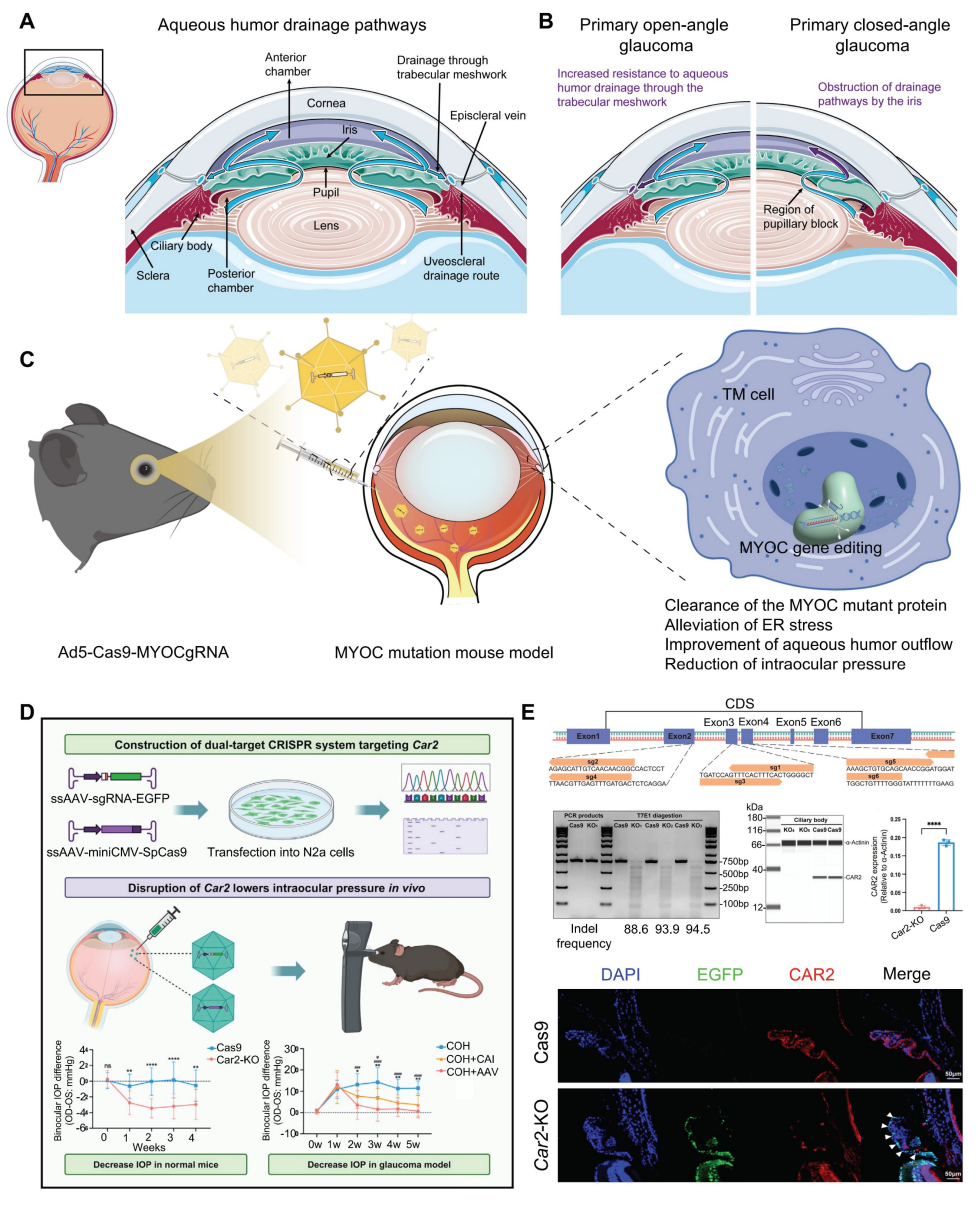
** Display of key results from recent glaucoma research: (A)** The aqueous humor circulation pathway. **(B)** The pathogenesis of glaucoma. **(C)** Using CRISPR/Cas9 technology to interfere with the *MYOC* gene *in vivo.* **(D-E)** AAV-mediated CRISPR/Cas9 *Car2* gene knockout for glaucoma treatment. Adapted with permission from 105, copyright 2024, Elsevier Inc.

Glaucoma is a progressive neuropathy primarily characterized by the degeneration of retinal ganglion cells (RGCs) and changes in the appearance of the optic disc, with elevated intraocular pressure (IOP) being the most notable clinical feature 101** (Figure 10A-B)**. Glaucoma exhibits a complex pathogenesis, with MYOC gene mutations representing significant genetic risk factors for this condition 102. Targeting this gene, Ankur Jain *et al.* delivered the CRISPR-Cas9 system *via* an adenoviral vector into the eyes of the *MYOC* mutant mouse model, which successfully knocked out the *MYOC* gene *in viv*o. This intervention alleviated endoplasmic reticulum stress within cells, significantly reduced IOP, and prevented further glaucoma damage in the mice's eyes 46** (Figure 10C)**. Meanwhile, BDgene Therapeutics has developed BD113, a therapy utilizing engineered lentiviral vectors to deliver gRNA/Cas9 ribonucleoprotein (RNP) complexes that target mutant *MYOC* gene for knockout. Currently, an ongoing single-dose, two-arm clinical study (ClinicalTrials.gov Identifier: NCT06465537), initiated by Beijing Tongren Hospital, aims to evaluate the efficacy of BD113 in treating primary open-angle glaucoma. Two BD113-treated patients exhibited no treatment-related serious adverse events, achieving normalized IOP and discontinuation of pressure-lowering medications.

Essentially, IOP is controlled from a structural abundance of aqueous humor production and outflow 103. Carbonic anhydrase, which regulates aqueous humor formation, has long been targeted for the treatment of glaucoma, yet local therapies face uveoscleral penetration/low bioavailability and systemic side effects 104. To address this issue, Zhang and colleagues recently proposed a CRISPR/Cas9 approach that directly targets the *Car2* gene in the ciliary body, using the serotype AAV vector ShH10, achieving a mean reduction of ~ 18% IOP in normal mice and ~ 40% IOP in a model of glaucoma 105** (Figure 10D-E)**. Importantly, CRISPR/Cas9 mediated *Car2*-KO remarkably alleviated RGC death and optic nerve fiber degeneration in a chronic high IOP model.

#### Congenital Aniridia

**Figure 11:**
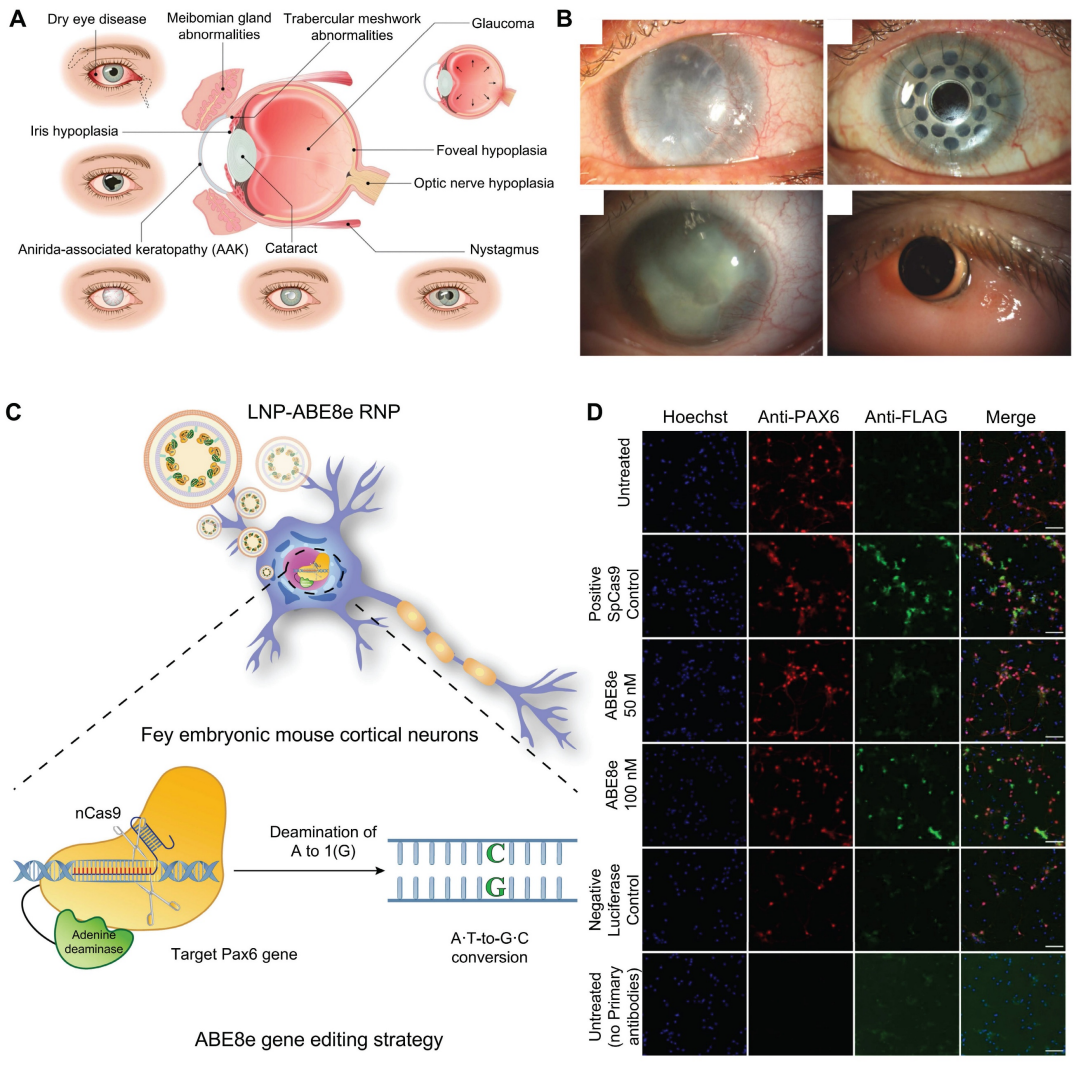
** Displaying the key findings of recent studies on congenital aniridia: (A)** Ocular manifestations of aniridia syndrome. Adapted with permission from 106, copyright 2023, Elsevier Inc. **(B)** Keratoprosthesis surgery in advanced aniridia. Adapted with permission from 106, copyright 2023, Elsevier Inc.** (C)** ABE8e strategy. **(D)** ABE8e strategy restores Pax6 expression. Adapted with permission from 110, copyright 2023, the authors.

Congenital aniridia is a rare genetic eye disorder characterized by varying degrees of iris and foveal hypoplasia 106 **(Figure 11A-B)**. This condition is inherited in an autosomal dominant pattern; mutations in the *Pax6* gene on the short arm of chromosome 11 (11p13) are responsible for roughly 90% of cases 107. Based on these mechanisms, using the CRISPR/Cas9 system, Roux and colleagues generated a *Pax6* gene mutation in human limbal stem cells as a cell model of aniridia-related corneal disease 108. The small eye (Sey) mouse carries a *Pax6* nonsense variant (c.580G>T, p.G194X), presenting with microphthalmia, aniridia, and other ocular phenotypes like human aniridia, making it a critical model for studying aniridia pathophysiology and therapies. Elizabeth et al. delivered an *in vitro*-optimized CRISPR/Cas9 system into Sey mutant zygotes *via* electroporation, successfully inserting a 3×FLAG tag into the Sey allele. This generated a novel mouse strain termed Fey, which retains the mutant characteristics of the Sey allele. The 3×FLAG tag enables researchers to distinguish wild type from edited Pax6 proteins, allowing precise evaluation of editing efficacy. Subsequently, the optimized CRISPR/Cas9 system was microinjected into Fey mouse zygotes, correcting the Sey mutation at 25% efficiency and restoring normal Pax6 expression. Genetically rescued mice exhibited wild-type ocular phenotypes 109.

ABE8e allows for the direct conversion from adenine (A) to guanine (G) in the genome and can obtain single-base editing without introducing DSBs. This strategy is milder than CRISPR/Cas9 technologies and minimizes nonspecific effects and potential off-target effects, and it is particularly appropriate for correcting specific point mutations responsible for the development of aniridia. Adair *et al.* encapsulated optimized ABE8e-RNP into lipid nanoparticle (LNPs) and delivered to Fey embryonic mouse cortical neurons, resulting in c.580G>T mutation correction and restoration of Pax6 protein levels (24.8%) based on this principle 110** (Figure 11C-D)**.

### Posterior Eye Disease

**Figure 12:**
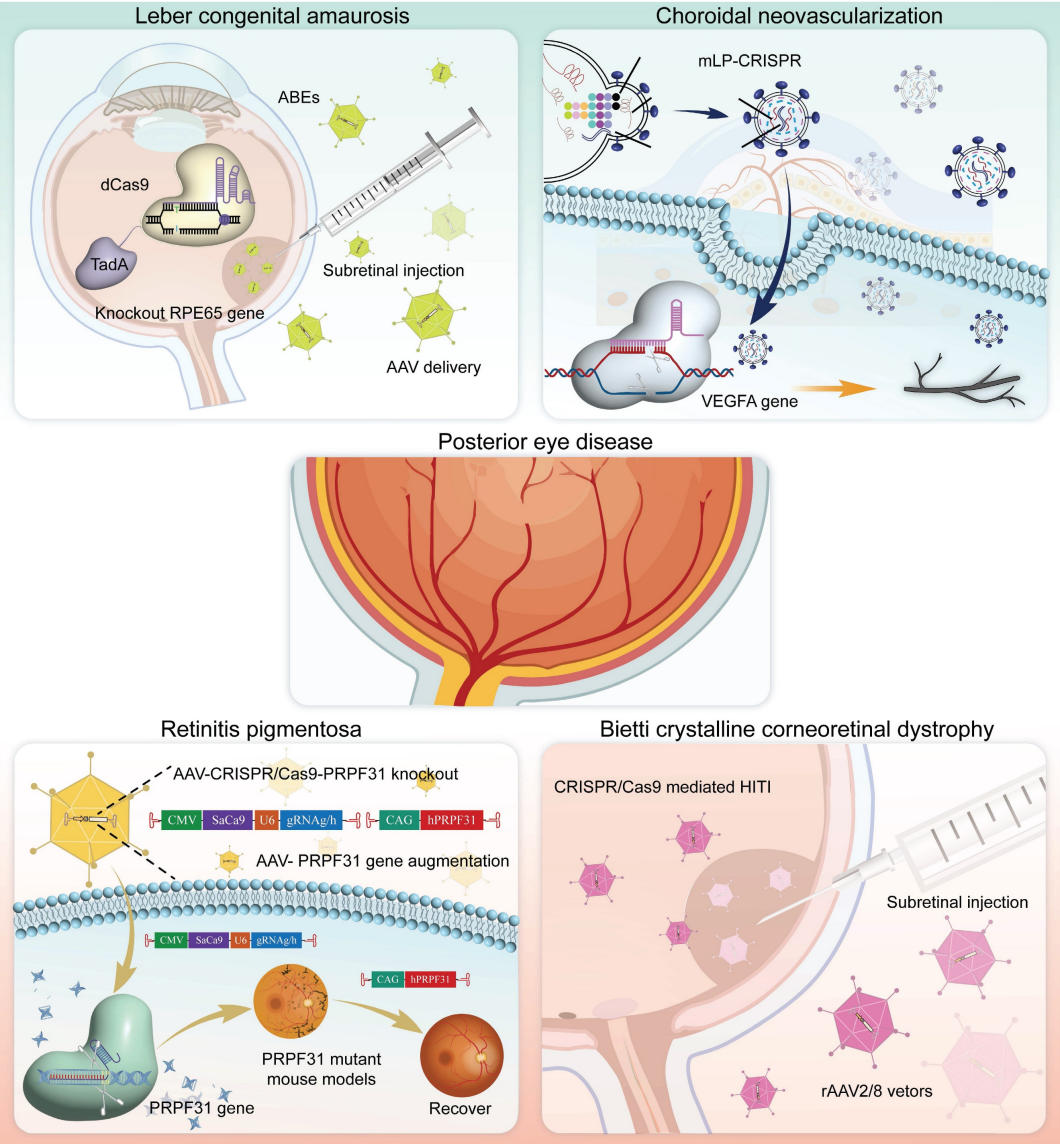
Representative applications of CRISPR system in posterior segment diseases.

The posterior segment of the eye, which is composed of intricate structures such as the retina, choroid, and optic nerve, is the core area for the transformation and transmission of visual signals. CRISPR technology, with its precise gene-editing capabilities, offers a revolutionary solution for correcting genetic defects, protecting neural cells, and promoting functional recovery. Below, we review the currently employed applications and advancements of CRISPR gene editing technology in the treatment of posterior segment diseases** (Figure 12)**.

#### Leber's Congenital Amaurosis (LCA)

**Figure 13:**
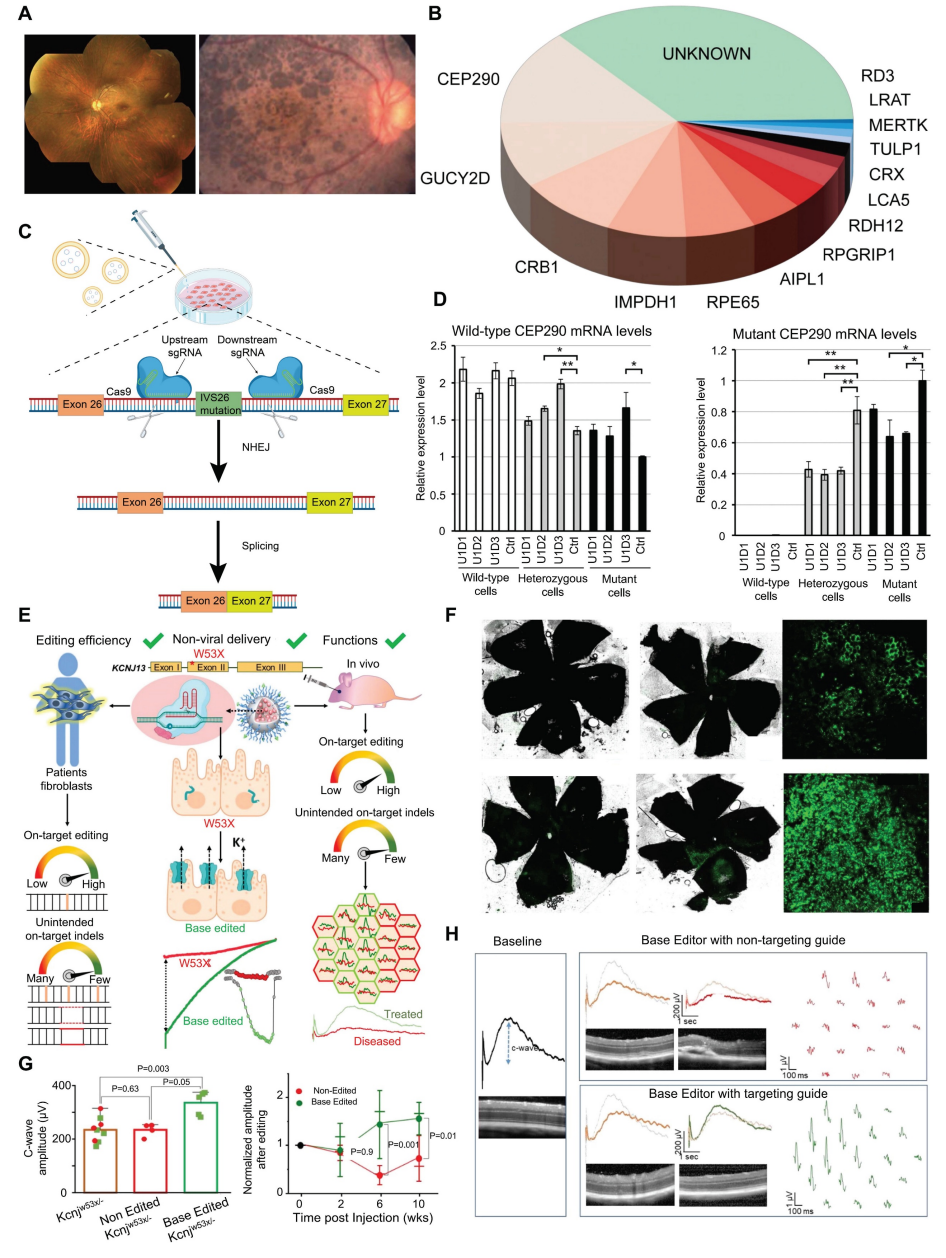
** Presentation of important data from recent studies on LCA, Part I: (A)** Fundus photography images of LCA patients. Adapted with permission from 120, copyright 2015, the authors.** (B)** The mutation genes associated with LCA. **(C)** The CRISPR/Cas9 strategy used to remove the IVS26 mutation*.* Adapted with permission from 115, copyright 2016, The American Society of Gene and Cell Therapy.** (D)** Rescue of the expression of WT *CEP290*. Adapted with permission from 115, copyright 2016, The American Society of Gene and Cell Therapy. **(E)** Using silica nanocapsules to deliver ABE8e to RPE cells for the treatment of inherited retinal diseases caused by *KCNJ13* gene mutations. Adapted with permission from 118, copyright 2023, Kabra *et al.* **(F-H)** ABE8e can effectively restore RPE cell function and improve retinal structure and function *in vivo*. Adapted with permission from 118, copyright 2023, Kabra *et al.*

LCA is a severe retinal dystrophy, usually manifesting at birth or in infancy, with progressive loss of vision. Clinical features of LCA can include nystagmus, sluggish pupillary response, night blindness, and severely reduced or absent ERG signals 111** (Figure 13A)**. Most LCA cases follow an autosomal recessive inheritance pattern and exhibit significant genetic and allelic heterogeneity 112. Now, more than 20 genes associated with LCA have been identified through genetic studies, including *CEP290*, *GUCY2D*, *CRB1*, *KCNJ13*, and *RPE65* **(Figure 13B)** 113. LCA10 is the most common subtype of LCA, caused by a deep intronic point mutation in intron 26 of the *CEP290* gene (IVS26 mutation) 114. Using the CRISPR/Cas9 system, two gRNAs were designed-one upstream and one downstream of the IVS26 mutation site-to direct the Cas9 nuclease in precisely excising the intronic fragment that contains the IVS26 mutation. Following deletion of the mutant region, the *CEP290* gene can undergo normal RNA splicing and produce full-length, functional CEP290 protein **(Figure 13C-D)** 115. Tailored for *CEP290*-associated IRDs such as LCA10, EDIT-101 is a CRISPR/Cas9-based gene editing therapy developed by Editas Medicine to excise the aberrant splice donor generated by the IVS26 mutation and thereby restore normal CEP290 expression. Subsequently, phase 1-2, open-label, single-ascending-dose study (ClinicalTrials.gov ID, NCT03872479) results demonstrated that 9 out of 14 patients treated with EDIT-101 showed meaningful improvements from baseline in the best corrected visual acuity. No treatment-related serious adverse events or dose-limiting toxicities were observed 116.

*KCNJ13* is critical for retinal pigment epithelium (RPE) function and encodes a potassium channel protein that modulates the electrophysiological response of the RPE to light-induced alterations in subretinal potassium concentration 117. Kabra and colleagues confirmed that ABE8e had improved *KCNJ13* W53X mutation gene editing efficiency and reduced off-target effects in HEK293T cells and patient-derived-of fibroblasts. To further study *in vivo* editing effects, researchers delivered ABE8e mRNA to mouse eyes *via* silica nanocapsules. Results showed a significant increase in the C-wave amplitude of edited mice's retinal response, indicating restored Kir7.1 channel function and maintained retinal structure 118** (Figure 13E-H)**. Additionally, Yuki Kanzaki *et al.* generated *KCNJ13* KO human induced pluripotent stem cells (hiPSCs) *via* the CRISPR/Cas9 system to successfully establish an LCA16 cell model 119.

**Figure 14:**
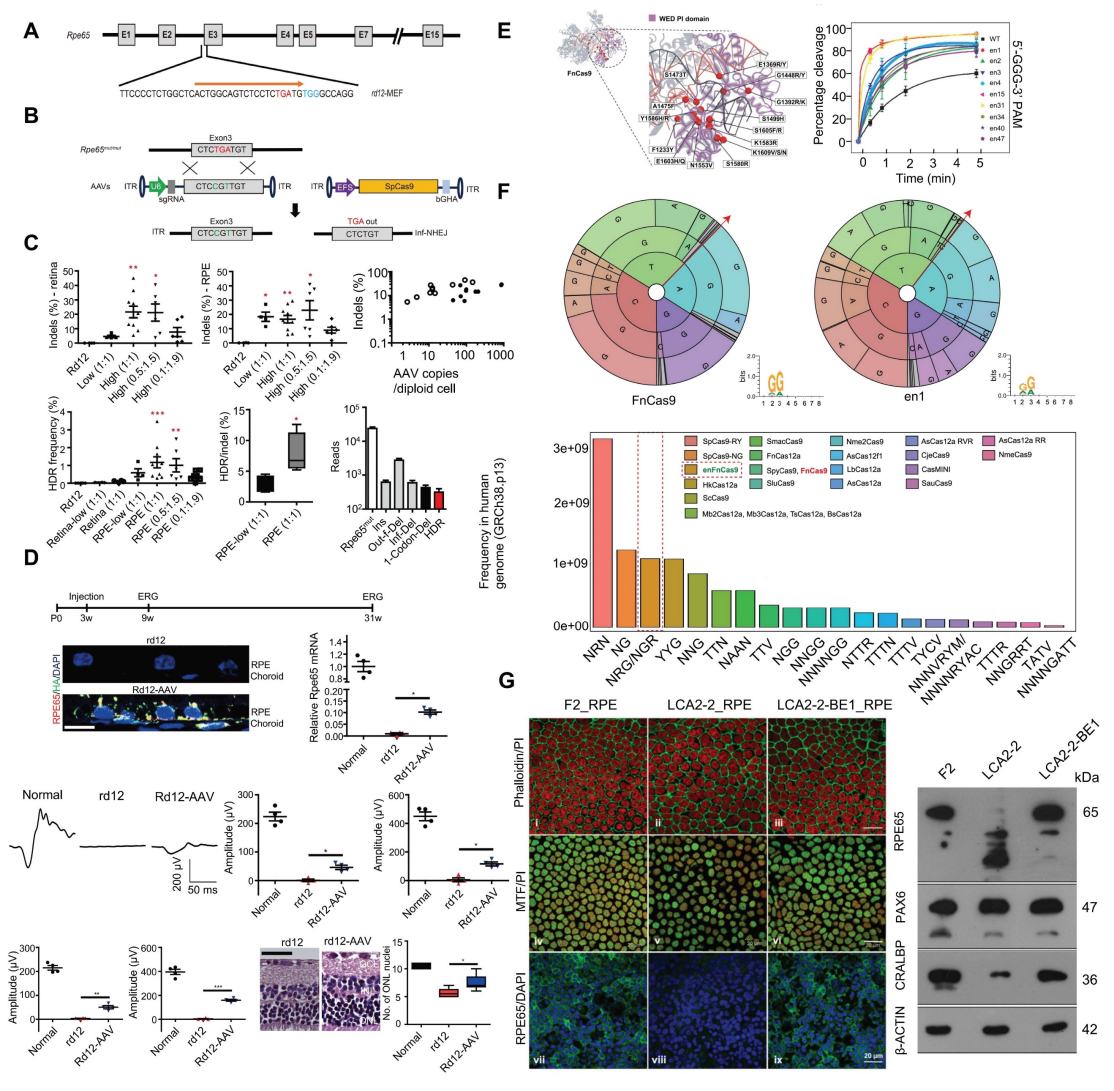
** Presentation of important data from recent studies on LCA, Part II: (A)** The structure of the *RPE65* gene is shown. Adapted with permission from 121, copyright 2019, the authors. **(B)** The positions of the premature stop codon. Adapted with permission from 121, copyright 2019, the authors.** (C)** *RPE65* gene correction in rd12 mice. Adapted with permission from 121, copyright 2019, the authors.** (D)** Therapeutic effects of HDR-mediated correction of the *RPE65* gene in rd12 mice. Adapted with permission from 121, copyright 2019, the authors.** (E)** FnCas9 crystal structure and *in vitro* cleavage assay. Adapted with permission from 122, copyright 2024, the authors.** (F)** PAM wheels and sequence logos for FnCas9 and enFnCas9, with bar plots showing the genome-wide availability of PAMs for each Cas effector in the human genome. Adapted with permission from 122, copyright 2024, the authors. **(G)** Therapeutic base editing by en31-ABEmax8.17d in a LCA patient specific iPSC line. Adapted with permission from 122, copyright 2024, the authors.

*RPE65* encodes a key isomerase that participates in generating the chromophore of cone and rod visual pigments. Loss of translation due to mutations in *RPE65* leads to premature termination of the translation process, which is one of the major causes of LCA. Jo and colleagues used a dual AAV vector encoding CRISPR/Cas9, and an *RPE65* donor sequence to target exon 3 of *RPE65* leading to the removal of the premature stop codon that caused LCA and restored retinal function in rd12 mouse model 121** (Figure 14A-D)**. In addition, Acharya's team altered FnCas9 to make three enFnCas9 variants (en1, en15 and en31). These possess increased editing efficiency and wider PAM specificity** (Figure 14E-F)**. They offer potential for gene editing and disease treatment, including the correction of *RPE65* mutations and restoration of pigment expression in RPE cells, which appear to be possible strategies for LCA treatment 122** (Figure 14G)**. Furthermore, Retinal injections were performed in mice using a lentivirus expressing ABE and sgRNA targeting the *de novo* nonsense mutation in the *RPE65* gene by Susie Suh and colleagues. Results showed a restored expression of RPE65 and vitamin A isomerase activity, as well as retinal and visual function approaching the normal range 123. Jang *et al.* delivered PE *via* AAV vectors to the subretinal space of rd12 mouse models, successfully corrected the nonsense mutation in the *RPE65* gene, effectively ameliorated disease phenotypes in the mice, and detected no off target editing 124.

Biallelic mutations in the *NMNAT1* gene are also an important cause of LCA 125. To investigate the mechanism by which *NMNAT1* mutations lead to retinal degeneration, during the differentiation of *NMNAT1*-knockout iPSCs created used CRISPR/Cas9 technology into retinal organoids, the formation of the retinal primordial structure failed, confirming the important role of *NMNAT1* in early retinal development 126. Since the *NMNAT1* gene mutation is recessive, supplementing the retina with a normal copy of *NMNAT1* has become a means to protect vulnerable cells from the effects of disease progression. Scott and colleagues performed gene augmentation therapy by subretinal injection of AAV carrying a normal human copy of *NMNAT1* in a mouse model harboring the *NMNAT1-p.Val9Met* mutation, rescuing retinal structure and function 127. To determine which cell types need to express *NMNAT1* for therapeutic effects, they treated *NMNAT1* mutant mice with AAV driven by specific cell type promoters and found that therapy promoting *NMNAT1* expression in photoreceptors could protect retinal morphology, suggesting that gene therapy for *NMNAT1* related diseases should target photoreceptors 128.

#### Retinitis Pigmentosa (RP)

**Figure 15:**
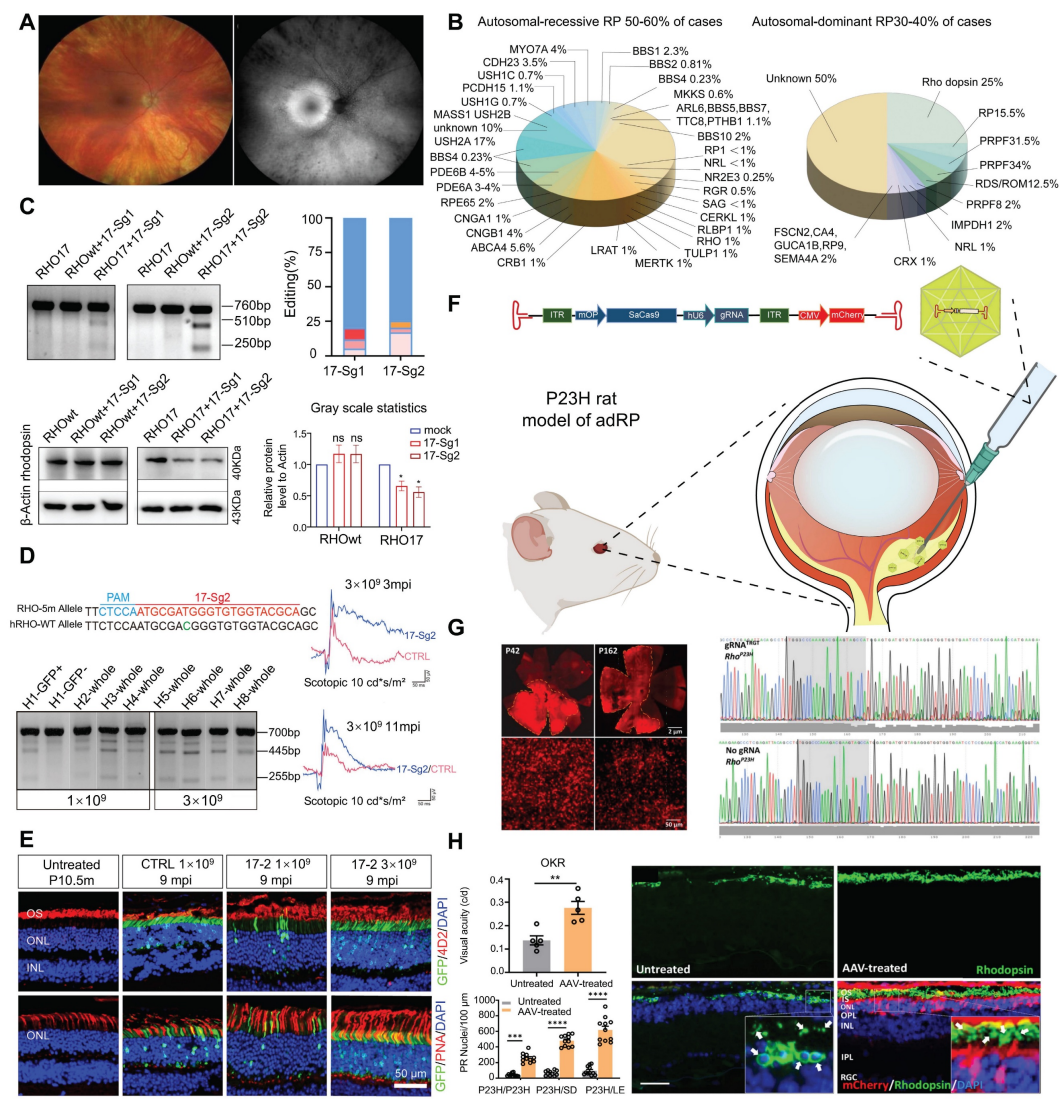
** Key data presentation of the latest achievements in RP, Part I: (A)** Fundus photography images of RP patients. Adapted with permission from 136, copyright 2023, the authors. **(B)** The currently known genes associated with RP and their relative contributions. **(C)** SaCas9/17-Sg1 and -Sg2 can specifically cut the mutant *RHO*-T17M sequence. Adapted with permission from 132, copyright 2023, Liu *et al.*** (D)** SaCas9/17-sg2 improves retinal function by editing mutant *RHO* gene. Adapted with permission from 132, copyright 2023, Liu *et al.* **(E)** The photoreceptor cells of *RHO* mutation mice treated with SaCas9/17-sg2 were preserved. Adapted with permission from 132, copyright 2023, Liu *et al.*** (F)** AAV-CRISPR/SaCas9 gene editing in the P23H rat model of adRP. **(G)** The CRISPR/Cas9 knocks out m-*Rho*P23H. Adapted with permission from 133, copyright 2022, the authors. **(H)** CRISPR/SaCas9-mediated ablation of m-*Rho*P23H protects photoreceptor cells. Adapted with permission from 133, copyright 2022, the authors.

RP refers to a class of inherited retinal dystrophies in which the rod photoreceptors and RPE undergo progressive degeneration, resulting in photoreceptor cell death and vision loss 129** (Figure 15A)**. RP can be inherited through autosomal recessive (50-60 %), autosomal dominant (30-40 %), and X-linked recessive (5-15 %) patterns **(Figure 15B)** 130. *RHO* gene have been recognized as a leading cause of autosomal dominant RP (adRP) 131. Liu *et al.* developed an allele-specific CRISPR/Cas9 gene editing therapy capable of selectively targeting the mutant *RHO*-T17M allele while preserving wild-type function **(Figure 15C-D)**. Through subretinal injection delivery into a humanized mouse model, it effectively delayed retinal degeneration and restored visual function 132** (Figure 15E)**. Saba Shahin and coworkers developed an AAV2-SaCas9/gRNA system effectively knocked out the *RHO* S334ter site, suppressing the P23H mutation. Through subretinal injection into P23H mutant rat models, this system delayed apoptosis of photoreceptors, and maintained long term vision and retinal function 133** (Figure 15F-H)**. Latella *et al.* delivered CRISPR/Cas9 plasmids designed to edit the human mutant *RHO* gene into mouse retinas *via* subretinal electroporation, achieving targeted gene editing and a significant reduction in mutant RHO protein levels. Notably, the engineered sgRNA exhibited high specificity for the human *RHO* gene without affecting the endogenous mouse *RHO* gene 134. Li and coworkers developed the engineered Cas9 variant and truncated sgRNA to achieve efficient discrimination of a single-nucleotide mutation in *RHO*-P23H mice. They achieved approximately 45% editing of the mutant P23H allele at the DNA level, while the editing rate of the wild-type allele was only 1.3% and the thickness of the photoreceptor cell layer in the treated retinal regions significantly increased. This study accomplished allele-specific editing of single-nucleotide mutations, expanding the application scope of CRISPR/Cas9 in the treatment of dominant genetic diseases 135.

Although CRISPR therapies targeting the specific P23H mutation have achieved numerous breakthroughs, developing separate treatments for over 150 distinct *RHO* mutations is not only costly but also clinically impractical. Wu *et al.* developed a CRISPR-based, mutation-independent gene ablation and replacement (AR) compound therapy, providing a universal solution for *RHO*-related adRP. This work overcomes the challenge of genetic heterogeneity in treating autosomal dominant disorders and holds direct promise for clinical transformation 137. Additionally, Du and colleagues utilized CRISPR/SaCas9-mediated delivery of dual AAV vectors for a “reduction and replacement” system to specifically silence the mutant *RHO* gene, while driving expression from the normal *RHO* allele 138.

CRISPRi inhibits dominant pathogenic gene expressions for avoiding the aggregate of toxic proteins, nudging cell longevity. Burnight *et al.* constructed a CRISPRi system that targets to *RHO* mutation gene pro23his. After subretinal transduction of human retinal explants and transgenic Pro23his mutant pigs, this system effectively suppressed Rho protein expression, maintenance of outer nuclear layer thickness and slowed retinal degeneration 139.

ZVS203e is a novel CRISPR/Cas9 based gene editing product designed to target and silence the mutated *RHO* gene. The drug was granted orphan drug designation by the U.S. Food and Drug Administration (FDA) in July 2022. To date, a single-arm, open-label Phase I/II clinical trial is currently underway to evaluate the safety and efficacy of ZVS203e in treating RP caused by *RHO* gene mutations (ClinicalTrials.gov ID, NCT05805007).

In addition to *RHO* gene, *PRPF31* mutations are also a common cause of adRP. Xi *et al.* employed an AAV-mediated CRISPR/Cas9 KO system to successfully replicate the morphological and functional damage associated with RP in mice, demonstrating the successful establishment of an RP animal model. Additionally, AAV-mediated *PRPF31* gene enhancement restored retinal structure and function in an RP mouse model 140. This study not only creates a novel system for studying the pathogenesis of *PRPF31*-associated RP but also provides valuable experimental evidence for the *PRPF31* gene therapy. Amélie Rodrigues *et al.* also used an AAV-mediated *PRPF31* gene enhancement technology repaired *PRPF31* gene mutations and improved the survival rate of photoreceptor cells 141.

**Figure 16:**
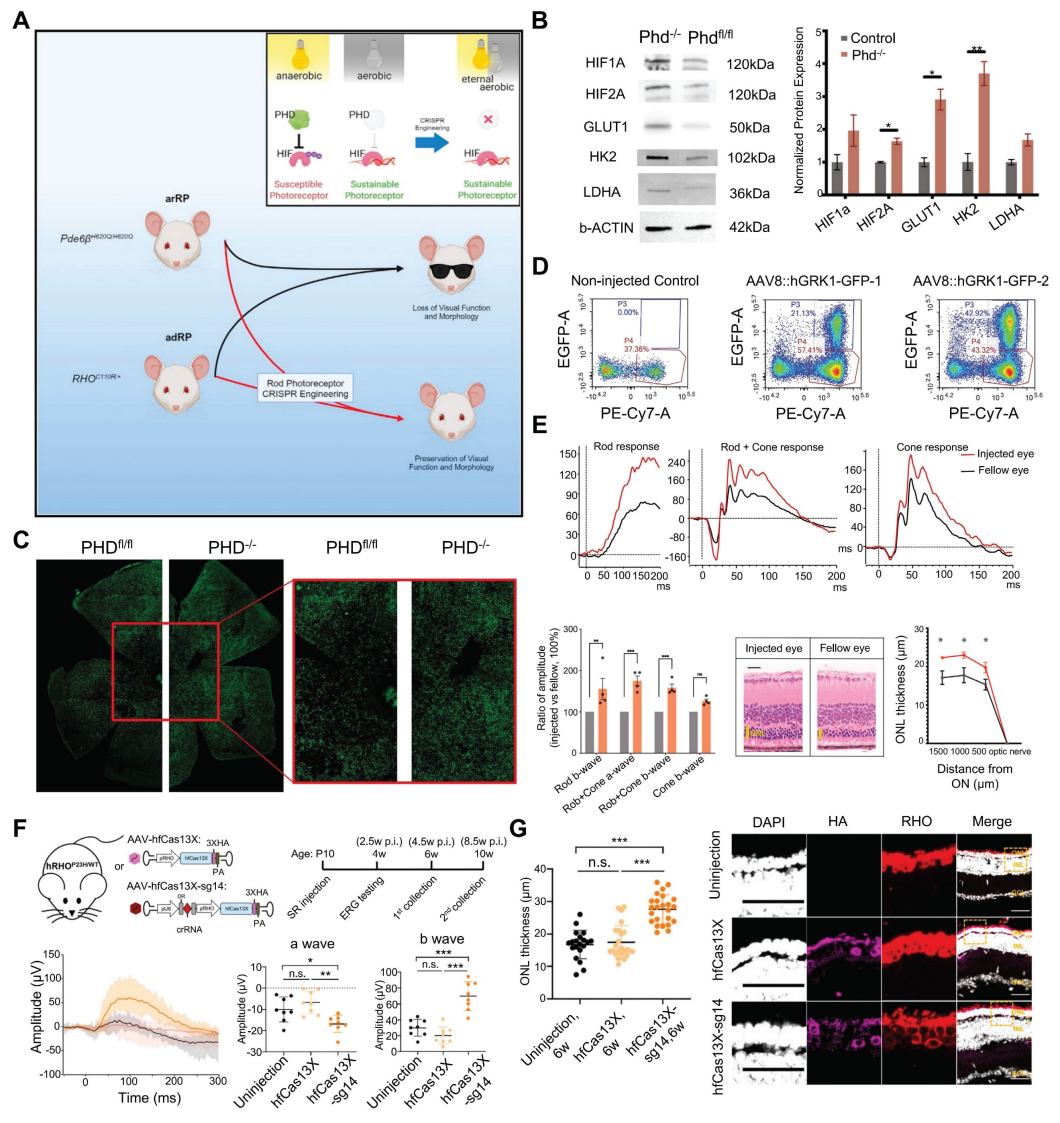
**Key data presentation of the latest achievements in RP research, Part II: (A)** CRISPR-mediated editing of the *PHD* gene for RP therapy. Adapted with permission from 142, copyright 2024, Elsevier Inc*.* **(B)** PHD deficiency up-regulates key regulators of glycolytic metabolism in photoreceptors. Adapted with permission from 142, copyright 2024, Elsevier Inc*.*** (C)** *PHD* knockout protects cone photoreceptors. Adapted with permission from 142, copyright 2024, Elsevier Inc*.*** (D)** AAV8 delivers hGRK1-GFP to mouse retinas. Adapted with permission from 142, copyright 2024, Elsevier Inc*.*** (E)** Detection of photosensitive function in RP mice. Adapted with permission from 142, copyright 2024, Elsevier Inc*.* **(F)** Strategy for treatment of RP mice with hfCas13X. Adapted with permission from 143, copyright 2023, the authors*.* **(G)** The protective role of AAV-hfCas13X for photoreceptor degeneration. Adapted with permission from 143, copyright 2023, the authors.

Recently, Nolan and colleagues developed a new dual gRNA-guided CRISPR/Cas9 system targeting exon 1 of the prolyl hydroxylase 2 (*PHD2*) gene based on the role of the PHD-von hippel lindau (VHL)-hypoxia inducible factor (HIF) axis in glycolysis **(Figure 16A)**. This dual gRNA-guided CRISPR/Cas9 system successfully knocked out the *PHD2* gene, promotes aerobic glycolysis and contribute to the survival of photoreceptor cells and improvements in retinal function 142 **(Figure 16B-E)**. This research represents a successful attempt to promote photoreceptor glycolysis through metabolic reprogramming *via* the CRISPR system, providing a novel perspective for treating this disease from a metabolic standpoint.

Cas13 editing of RNA offers a therapeutic approach that avoids the risks associated with permanent genomic changes. Cas13X degrades target RNA with high specificity and minimizes off-target effects, ensuring therapeutic efficacy while preserving nontarget RNA. Yan *et al.* designed a compact and high-fidelity Cas13X (hfCas13X) sgRNA CRISPR system targeting the human mutant ribozyme transcript *RHO*-P23H **(Figure 16F)**. This system was delivered by AAV injected into the vitreous cavity of RP mouse models, leading to retinal function improvement and inhibition of photoreceptor apoptosis and retinal thinning 143 **(Figure 16G)**.

#### Bietti Crystalline Corneal Retinal Dystrophy (BCD)

BCD is an autosomal recessive progressive degenerative disease of the chorioretina, which is characterized by abundant yellow-white crystalline deposits in the retina, particularly around the posterior pole 144. Currently, *CYP4V2* is the only gene identified to be associated with BCD 144. Given that there are no approved treatments for BCD, researchers are increasingly exploring CRISPR-based therapies for this genetic condition. Reports indicate that approximately 80% of BCD patients have mutations in exons 7-11 of the *CYP4V2* gene 144. HITI is a precise gene editing technique that enables the insertion of DNA sequences at specific loci without relying on homologous recombination 48. Meng *et al.* created a sgRNA to direct the Cas9 nuclease to make DSBs in intron 6 of the *CYP4V2* gene and used the HITI method to successfully insert the sequence carrying exons 7-11 at the intron 6 site of the *CYP4V2* gene. In patient-derived iPSCs, the HITI method revitalized the viability and functionality of RPE cells. In a humanized *Cyp4v3* mouse model, this strategy successful increased numbers of RPE cells, metabolic activity and significant improved retinal function postediting 50. CRISPR/Cas9-mediated HITI has a promising application to those IRDs genes with mutation hotspots and can serve as an effective complement to gene replacement therapy.

#### Stargardt Disease (STGD1)

STGD1 is currently an untreatable autosomal recessive macular dystrophy primarily caused by biallelic mutations in the *ABCA4* gene 145. The most prevalent mutation associated with STGD1 is a G-to-A point mutation in *ABCA4* (c.5882G>A, p. Gly1961Glu), affecting approximately 15% of patients 146. An ABE strategy utilizing AAV vectors was developed to correct the *ABCA4* c.5882G>A mutation. High-level gene correction was achieved in mutation-carrying mice and nonhuman primates, with mean editing rates of 75% in cone photoreceptors and 87% in RPE cells. However, functional improvements following editing could not be validated due to the absence of disease phenotype in animal models 147.

Moreover, several deep intronic variants (DIVs) in *ABCA4* have been identified as pathogenic for STGD1, such as c.5197-557G>T, which causes aberrant splicing, resulting in the generation of a premature termination codon 148. Angeli *et al.* designed three CRISPR/Cas9 approaches (sgRNA/SpCas9, dual gRNAs/SpCas9, and dual gRNAs/SpCas9 nickase) to address this splicing defect. In patient-derived cone photoreceptor precursor cells carrying the c.5197-557G>T variant, these strategies were able to restore correct splicing by up to 83% and increase the total amount of correctly spliced transcripts by 1.8-fold 149. This study demonstrates that CRISPR/Cas9 technology can effectively repair splicing defects caused by *ABCA4* DIVs, providing a novel therapeutic strategy for STGD1.

#### Retinal Neovascularization

**Figure 17:**
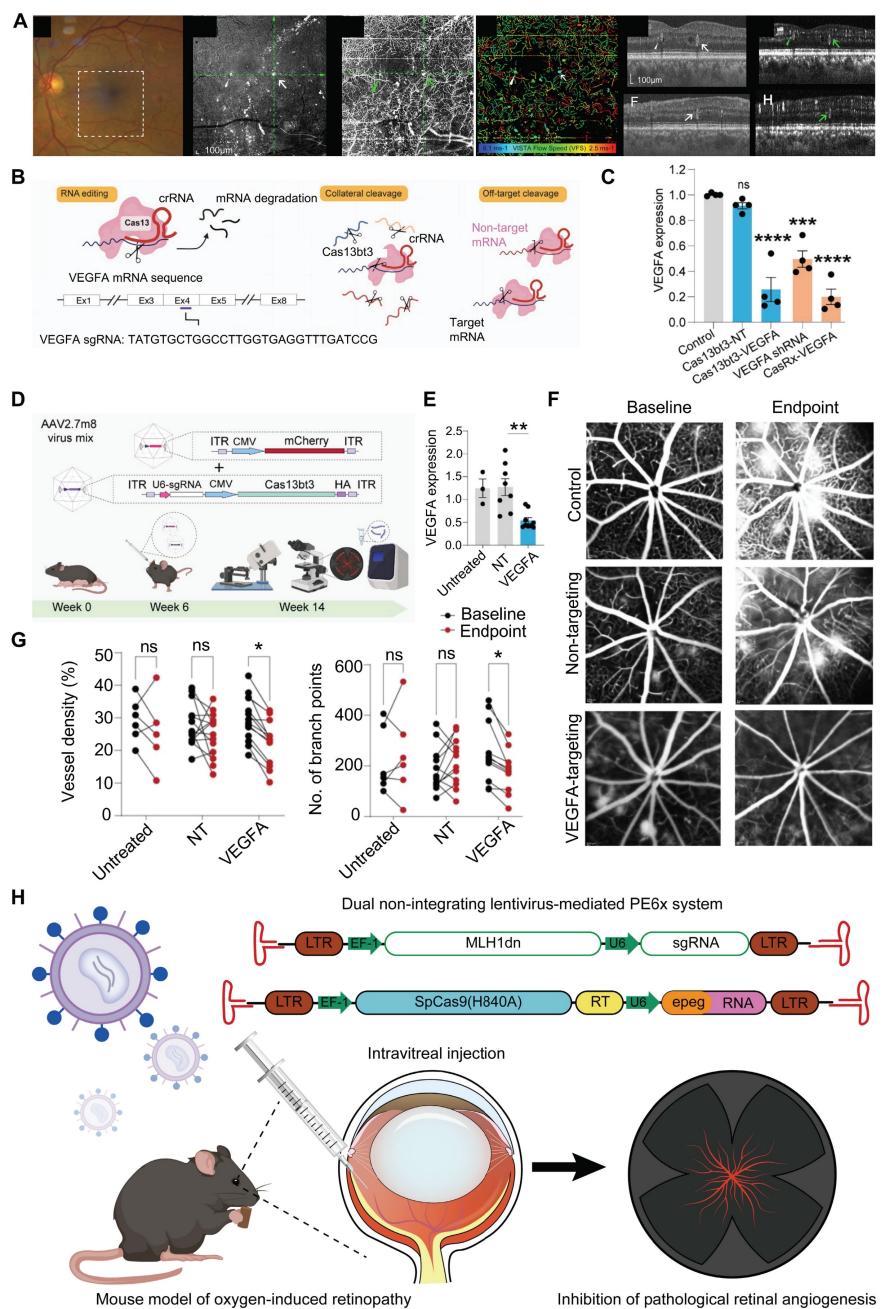
** Recent key research achievements in retinal neovascularization: (A)** Multimodal imaging of a patient with diabetic retinopathy imaged by highspeed, swept-source OCT. Adapted with permission from 152, copyright 2025, the authors.** (B)** Schematic of single and multiplex targeting with Cas13bt3. Adapted with permission from 155, copyright 2024, the authors. **(C)** VEGFA mRNA knockdown from Cas13bt3. Adapted with permission from 155, copyright 2024, the authors.** (D)** Schematic of experimental procedure for intravitreal treatment of Kimba mice. Adapted with permission from 155, copyright 2024, the authors.** (E)** Expression of human VEGFA mRNA in Kimba retinas. Adapted with permission from 155, copyright 2024, the authors.** (F)** Central fluorescein fundus angiography images after Cas13bt3 treatment. Adapted with permission from 155, copyright 2024, the authors.** (G)** Evaluate the situation of angiogenesis. Adapted with permission from 155, copyright 2024, the authors.** (H)** Schematic of PE6x system-mediated suppression of angiogenesis in an oxygen-induced retinopathy mouse model.

The retinal structure is morphologically divided into ten layers, with the inner five layers receiving blood from the central retinal artery and its branches 150. Hypoxia and the accumulation of metabolic byproducts in the retina often leads to the development of retinal neovascularization 151. This condition is not classified as a distinct disease; rather, it is closely associated with various systemic diseases and retinal disorders, such as diabetic retinopathy and retinopathy of prematurity 152, 153** (Figure 17A)**. Clinically, treatment for retinal neovascularization mainly involves anti-VEGF antibodies (such as ranibizumab) or antagonists of the VEGFR2 (such as aflibercept) 154. However, both approaches require repeated long-term administration, imposing significant economic and lifestyle burdens on patients and presenting substantial challenges. CRISPR technology may offer a promising new solution to these issues.

CRISPR/Cas13-based Cas13bt3 gene therapy can effectively silences VEGFA mRNA in both human retinal organoids and humanized VEGF transgenic mouse models **(Figure 17B-C)**. Intravitreal delivery of Cas13bt3 by AAV2.7m8 significantly inhibits retinal neovascularization in the mouse 155 **(Figure 17D-G)**. Cas13bt3 is smaller (∼775 amino acids) compared to traditional CRISPR/Cas13 systems, which allows it to be delivered *via* AAV vectors successfully 68. With further preclinical research and optimization, Cas13bt3 is expected to become an effective gene therapy tool for treating retinal neovascular diseases.

VEGFR2 mediates almost all known VEGF-induced effects, including its effects on increasing microvascular permeability and angiogenesis 156. Addressing this mechanism, Wu *et al*. developed an AAV-mediated CRISPR/SpCas9 system targeting *VEGFR2*. This system was delivered into retinal vascular endothelial cells in a mouse model of oxygen-induced retinopathy *via* intravitreal injection, enabling *VEGFR2* genome editing and suppressing pathological retinal neovascularization 157. Additionally, Huang* et al.* established a PE6x system within two lentiviral vectors, with one carrying an enhanced PE gRNA and Cas9 nickase fused with an optimized reversal transcriptase, and the other conveying a nicking gRNA and a dominant negative DNA mismatch repair protein (DN-MLH1) to improve PE efficiency 158. The PE6x system was employed to introduce a T17967A mutation at the *VEGFR2* gene locus, which resulted in the generation of an earlier stop codon (TAG, K796stop) from the original AAG (K796). Because of the PE6x induced stop codon, a dominant negative VEGFR2 (DN-VEGFR2) with 795 amino acids was produced during translation. This DN-VEGFR2 effectively blocked VEGF induced VEGFR2 phosphorylation and inhibited the activation of the VEGF/VEGFR2 signaling, thereby impeding pathological retinal neovascularization 158** (Figure 17H)**. Unlike traditional gene editing methods, PE6x allows for the various gene modifications, such as base substitutions and small insertions/deletions, to be performed without generating a DSB. This greatly minimizes off-target effects and potential genomic instability.

#### Choroidal Neovascularization (CNV)

**Figure 18:**
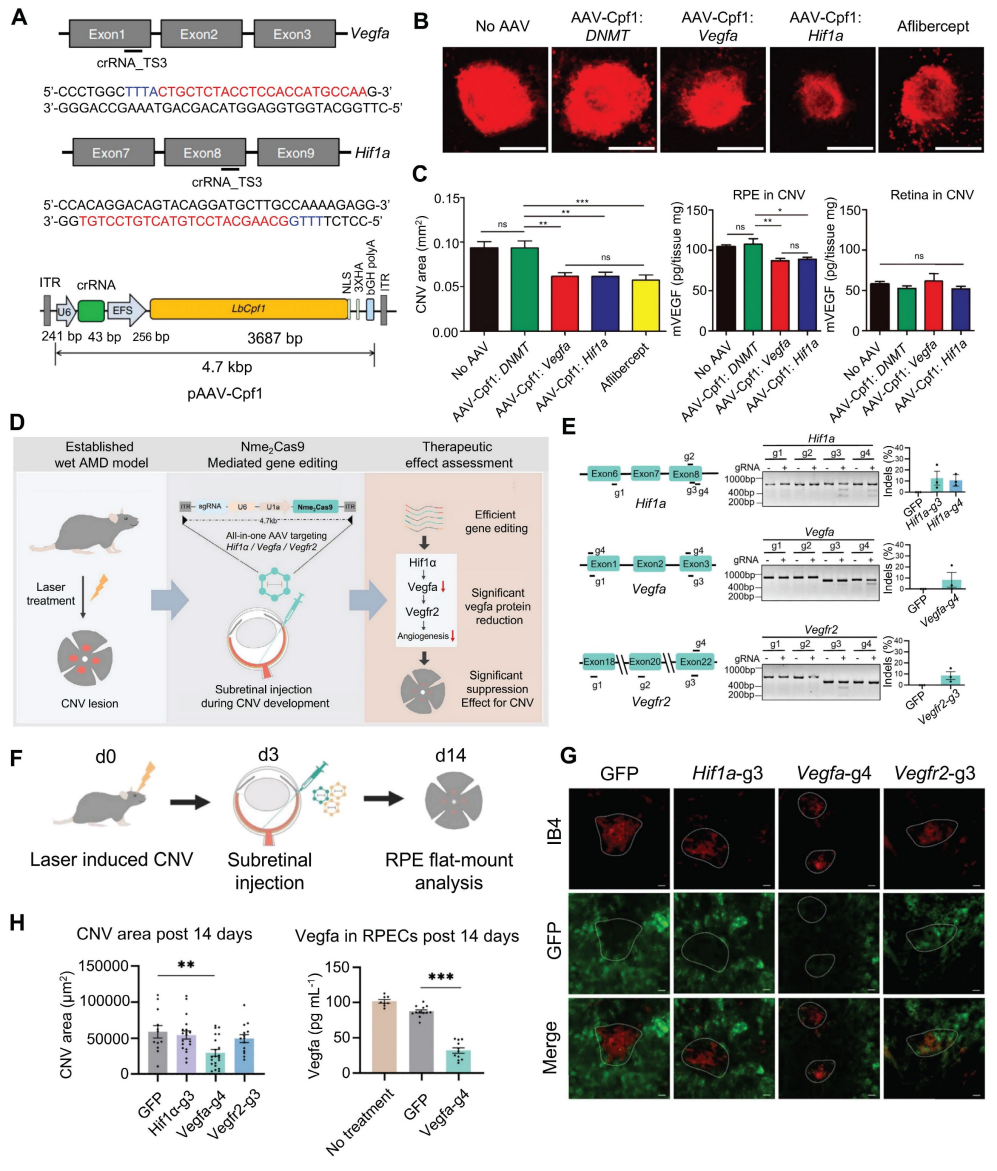
**Summary of recent representative research results on CNV: (A)** LbCpf1 crRNA targets *Vegfa* and *Hif1a* genes. Adapted with permission from 161, copyright 2018, the authors.** (B-C)** LbCpf1 gene editing can reduce the area of laser-induced CNV. Adapted with permission from 161, copyright 2018, the authors.** (D)** Nme2Cas9 gene editing can significantly alleviate CNV*.* Adapted with permission from 162, copyright 2023, John Wiley & Sons Australia, Ltd.** (E)** Nme2Cas9 sgRNA targets *Hif1α*, *Vegfa, Vefgr2*. Adapted with permission from 162, copyright 2023, John Wiley & Sons Australia, Ltd.** (F-H)** AAV-delivered Nme2Cas9 knocks out *Vegfa*, inhibiting laser-induced CNV. Adapted with permission from 162, copyright 2023, John Wiley & Sons Australia, Ltd.

CNV is defined as new blood vessels that proliferate from the choroid and protrude through fenestrations in Bruch's membrane into the subretinal pigment epithelium or subretinal space 159. So far, studies on CRISPR gene editing of CNV are strongly biased towards traditional pro-angiogenic elements such as VEGFA 160. Utilizing the AAV9 vector to deliver the crRNA-guided endonuclease LbCpf1 derived from Lachnospiraceae bacteria, Taeyoung Koo and colleagues targeted the retinas of mice to knockout the angiogenesis-related genes *Vegfa* and *Hif1α* 161** (Figure 18A)**. The coding sequence of LbCpf1 (~3.7 kbp) is less than that of SpCas9, enabling its packaging into AAV vectors for delivery. Importantly, targeting *Vegfa* or *Hif1α* with LbCpf1 significantly reduced the area of laser-induced CNV 161** (Figure 18B-C)**.

Nme2Cas9 is a Neisseria meningitidis-derived Cas9 protein. Compared with SpCas9, Nme2Cas9 is smaller in size and can be packaged into a single AAV vector. Hu *et al.* designed sgRNAs targeting the *Hif1α*, *Vegfa*, and *Vegfr2* genes and delivered the AAV-mediated Nme2Cas9/sgRNA system to mouse RPE cells *via* subretinal injection **(Figure 18D-F)**. The results demonstrated that only early intervention with the *Vegfa* gene significantly reduced the area of CNV lesions (by 49.5%), whereas intervention with *Hif1α* and *Vegfr2* did not yield significant therapeutic effects 162** (Figure 18G-H)**.

Moreover, Park and colleagues previously substituted the CMV promoter with an RPE-specific one (VMD2) in a lentiviral vector expressing Cas9, resulting in specific Cas9 expression and high editing efficiency in RPE cells 163. The VMD2 driven gVEGFA/Cas9 system knockout effectively inhibited laser-induced CNV, with little adverse effects on the neural retina, providing a new strategy for the safer and effective treatment of CNV 163. Ling and colleagues synthesized an integrated CRISPR lentiviral vector (mLP-CRISPR) carrying both Cas9 and gRNA.

This mLP-CRISPR system can effectively inhibit the formation of CNV in a laser-induced mouse model 164. Notably, the mLP-CRISPR system does not elicit potent innate or adaptive immune responses during *in vivo* applications, with reduced potential safety risks. HG202 developed by HuidaGene Therapeutics is a CRISPR/Cas13-based RNA editing candidate for the treatment of nAMD, and it works by locally reducing the expression of VEGFA mRNA within the retina. Currently, HG202 is undergoing clinical trials to evaluate its safety, tolerability, and efficacy (ClinicalTrials.gov ID, NCT06031727).

#### Retinoblastoma

Retinoblastoma (RB), the predominant pediatric retinal malignancy, arises from biallelic inactivation of the chromosome 13-located tumor suppressor gene *Rb1* in germline or somatic lineages 165. Although recent advances have improved RB cure rates, current treatments still lack effective molecular targeted drugs, partly because of the preclinical research is limited by existing animal models characterized by long tumor latency and low penetrance 166. Pioneering this frontier, Naert *et al*. engineered CRISPR/Cas9 mediated co-knockout of *Rb1* and retinoblastoma-like 1 (*Rbl1*) in Xenopus tropicalis, establishing the non-mammalian genetic RB model exhibiting rapid-onset tumors (minimum 36 days) and high penetrance (73%) 167. This paradigm provides an ethically and economically optimized platform for deconvoluting RB pathogenesis and accelerating drug discovery. Notably, 6% of heritable RB cases progress to trilateral retinoblastoma (TRB), characterized by synchronous intracranial neuroectodermal malignancies 168. Leveraging human embryonic stem cells (hESCs) as a developmental model, Avior *et al.* established *Rb1*-null hESCs *via* CRISPR/Cas9 editing. These cells generated teratomas exhibiting neural overproliferation and mitochondrial dysfunction-key features of TRB pathology 169. This model elucidates both the developmental roles of *Rb1* and its tumorigenic effects, providing a robust platform for drug discovery.

## CRISPR Delivery Systems

**Figure 19:**
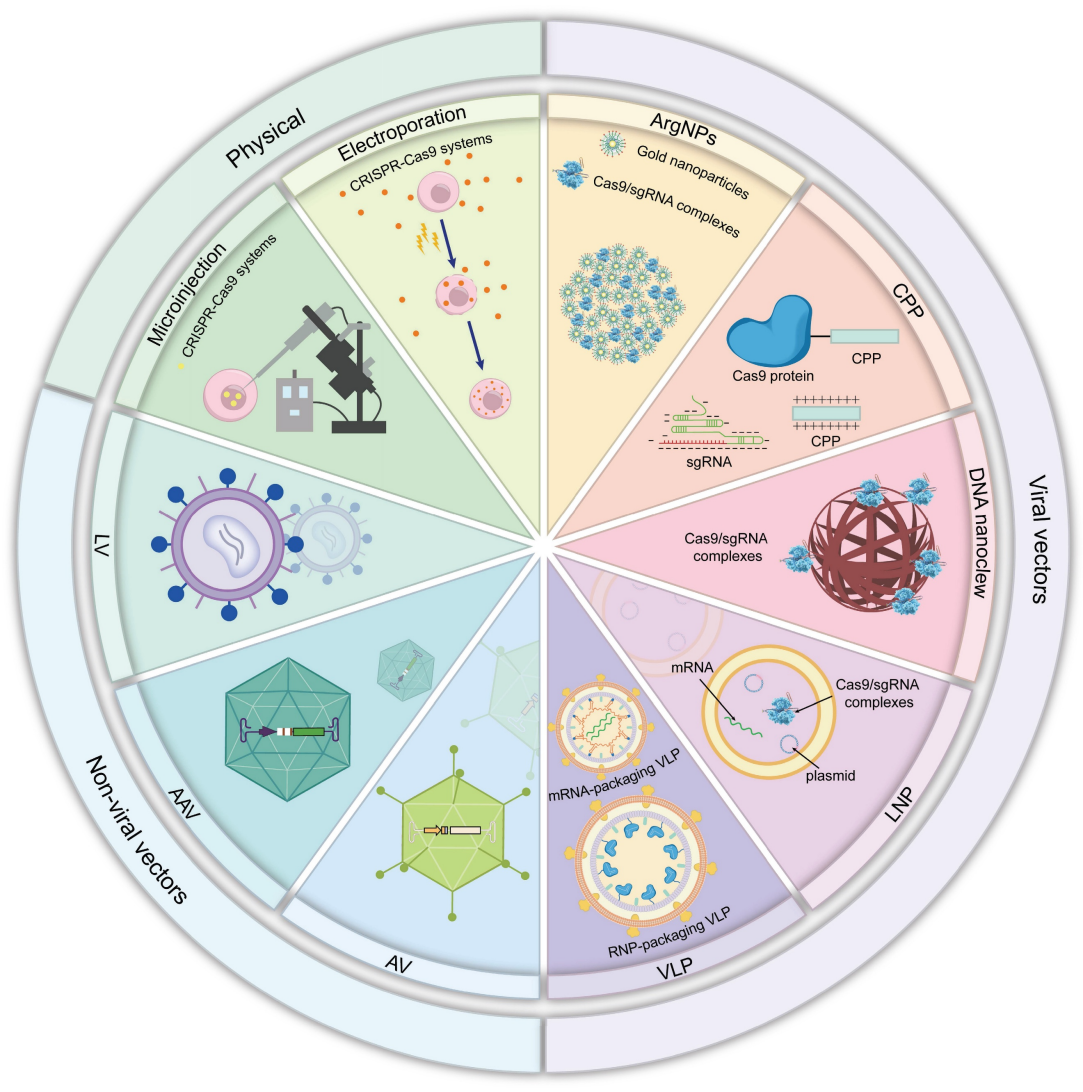
Schematic diagram of CRISPR delivery vectors.

The development of gene editing technologies, particularly that of the CRISPR/Cas9 system, has opened new avenues for the treatment of various genetic disorders 171, 172. However, the effective delivery of these systems to target cells *in vivo* necessitates the development of suitable delivery strategies. In recent years, substantial advancements have been made in delivery methods for CRISPR systems, evolving from early viral vectors and physical delivery to the current extensively researched LNPs and virus-like particles (VLPs) 173 **(Figure 19)**.

The physical delivery methods of the CRISPR system involve directly penetrating the cell membrane or cell wall through physical means, such as electroporation and microinjection, to deliver CRISPR components into cells 174. Viral vector delivery systems, including AAVs, lentivirus (LVs) and adenovirus (AVs), have been widely studied because of their high transduction efficiency. AVs leverage high transduction efficiency and large cargo capacity to mediate transient gene expression, making them suitable for vaccine development and large-fragment gene repair 175. AAVs enable safe and targeted delivery due to their low immunogenicity, sustained expression, and serotype-dependent tissue tropism, establishing AAVs as the most validated and widely deployed platform for *in vivo* gene delivery 176. LVs enable long-term stable expression through genomic integration capability, infect both dividing and non-dividing cells, and are suitable for establishing stable cell lines and long-term *in vivo* editing 177. However, the limited payload capacity of viral vectors and potential for eliciting immune responses limit their application.

These disadvantages of AAV vectors have led to the exploration of non-viral delivery systems, which include mainly cell-penetrating peptide (CPPs), gold nanoparticles (AuNPs), DNA nanoclew, LNPs and VLPs 178. Non-viral delivery methods have many important benefits such as their ability to avoid the limitations of viral packaging sizes and a greatly increased ability to tightly control the dosage, duration, and specificity of action 179. Of these, LNPs are the most well representative non-viral delivery vehicles to date. Typically, negatively charged nucleic acids form complexes with positively charged lipids through electrostatic interactions, creating LNPs that protect the nucleic acids from nuclease degradation and facilitate cellular uptake *via* endocytosis. By optimizing lipid formulations, LNPs achieve efficient and low-toxicity targeted delivery to specific tissue cells 180. VLPs are self-assembled empty shells composed of viral structural proteins, capable of efficiently delivering mRNA and RNP to specific cells 181. Combining viral transduction efficiency with the biosafety of non-viral vectors, VLPs have emerged as a promising delivery platform for CRISPR-based therapy. Additionally, CPPs enable carrier-independent delivery of CRISPR systems *via* short peptide mediated efficient transmembrane transport 182; AuNPs possess dual capabilities for multiplexed cargo loading and photothermal/photo-triggered release 183; DNA nanoclew utilizes rolling circle replication technology to self-assemble into high density DNA nanostructures, achieving efficient CRISPR RNP delivery 184.

In summary, the development and refinement of CRISPR/Cas9 systems are progressing rapidly, with an increasing number of studies focusing on optimizing their composition and delivery strategies to enable broader clinical applications** (Table 2)**.

## Safety and Ethical Regulations of CRISPR/Cas Gene Editing Systems

From 2025 onwards, there are going to be 10 to 20 gene therapies approved annually by the FDA, which indicative of the rapid development and increasing maturity of gene therapy 185. Luxturna, a gene augmentation therapy delivering functional RPE65 to RPE cells for treating LCA, marking a milestone in ophthalmic gene therapy 186. CRISPR gene editing technology overcomes the limitation of conventional gene replacement therapy, which only target autosomal recessive disorders, and significantly expanding the scope of gene therapy applications. Notably, multiple candidate drugs demonstrate significant clinical promise, such as the EDIT-101, HG202, ZVS203e, BD111 and BD113. These collective advances establish a robust foundation for future regulatory approvals of CRISPR-based ocular therapeutics.

Despite significant advancements in the precision of CRISPR technology, safety concerns such as off-target effects, immune responses, and the long-term stability of gene editing systems remain critical issues 187. Therefore, comprehensive preclinical studies and clinical trials to evaluate the safety and potential long-term impacts of these technologies are essential. Ophthalmic safety assessments should include the following:

(1) Behavioral tests such as localization tasks, alongside electrophysiological tests such as visual evoked potential, to evaluate changes in vision resulting from CRISPR gene editing.

(2) Optical coherence tomography (OCT) for obtaining high-resolution images of retinal structure, aiding in the assessment of retinal changes post-CRISPR treatment, including changes in thickness, layering, and structural integrity.

(3) Fundus fluorescein angiography (FFA) to observe the health of retinal blood vessels and identify leakage or pathological changes.

(4) Histological analyses to examine cellular morphology, structural changes, and expression of damage markers in ocular structures.

(5) Measurement of biomarkers of ocular inflammation (e.g., cytokines and chemokines) to evaluate the immune response elicited by gene editing technology.

By employing these methodologies, researchers can comprehensively assess the extent of damage and potential side effects of CRISPR gene editing technologies on the eye, thus providing crucial experimental data to support clinical applications.

The application of CRISPR technology has raised many ethical concerns. Therapeutic editing aims to repair or replace defective genes to cure diseases, whereas enhancement editing seeks to improve an individual's traits or capabilities. The ambiguity surrounding these boundaries can lead to ethical debates, such as whether gene editing should be permitted for healthy individuals to enhance vision or other characteristics. Furthermore, patient autonomy and informed consent are paramount in gene editing, particularly when it involves minors or individuals with limited cognitive ability; ensuring that their interests are adequately represented is a complex challenge.

To ensure the safe and responsible application of CRISPR technology, effective regulation is essential. Ethical review mechanisms concerning CRISPR applications need to be strengthened to promote transparency and compliance in research, including training for researchers and the formation of independent review boards. Regulatory agencies must establish clear guidelines and regulations to ensure both the safety and ethical integrity of the technology. As gene editing technologies such as CRISPR continue to evolve, the scientific community, ethicists, and policymakers must work together to formulate ethical guidelines that align with societal values.

## Ocular Diseases Remain Unaddressed by CRISPR/Cas Gene Editing Systems

CRISPR technology was initially applied primarily to treat rare blinding hereditary eye diseases such as LCA and RP. As the technology matures, CRISPR has begun to demonstrate promise in common ophthalmic conditions like AMD and viral keratitis. Nevertheless, numerous vision-threatening diseases still lack effective treatments. CRISPR offers new hope for managing these conditions.

(1) Keratoconus (KC): KC is a common progressive corneal disorder characterized by corneal thinning and asymmetric conical protrusion, which can cause severe visual impairment 188. The pathogenesis of KC is highly complex, involving interactions among genetic, environmental, and mechanical factors 189. Polygenic loci such as *ZNF469*, *VSX1*, and *SOD1* have been identified 190. CRISPR-assisted high throughput genetic screening can help uncover disease associated genetic variants, facilitating the discovery of novel genetic markers. The cornea's biophysical properties make it an ideal candidate for CRISPR therapy. For instance, CRISPRi technology could knockout matrix metalloproteinase-related genes (such as *Timp3*) to inhibit corneal stromal degradation, halting disease progression. Additionally, developing keratoconus animal models using CRISPR technology represents a promising avenue for research.

(2) High myopia: High myopia can lead to complications such as macular degeneration, retinal detachment, and glaucoma, representing a major cause of irreversible blindness in young adults 191. It is currently understood as a multifactorial disorder involving both genetic and environmental factors, with over 30 associated loci reported (including LRRC46*,* SCO2*,* CCDC111*,* SLC39A5*,* CPSF1) demonstrating a complex polygenic inheritance pattern 192. CRISPR technology shows significant research potential for high myopia. Specifically, using a CRISPR activation system to upregulate COL1A1 expression, delivered *via* AAV vectors targeted to scleral cells through posterior scleral injection, holds promise for enhancing scleral collagen cross-linking and slowing myopia progression.

(3) Dry eye disease (DED): DED is a multifactorial ocular surface disorder that significantly impairs patients' quality of life and severe cases may lead to corneal ulcers and perforation 193. Chronic inflammation represents a core mechanism in DED pathogenesis 194. CRISPR technology enables targeted modulation of key genes involved in ocular surface inflammation and immune responses 195. For instance, CRISPR/Cas9-mediated gene knockout can specifically reduce expression of pro-inflammatory cytokines (e.g., TNF-α, IL-1β) in ocular surface cells, thereby mitigating inflammatory cascades. For aqueous-deficient DED caused by lacrimal gland dysfunction, CRISPR-based overexpression of regenerative growth factors may promote repair and functional restoration of damaged lacrimal tissue.

(4) Congenital stationary night blindness (CSNB): CSNB is a group of inherited retinal disorders caused by dysfunctional signal transduction within the retina 196. To date, over 20 CSNB-associated pathogenic genes have been identified 196. While animal studies have successfully restored visual function in CSNB models using AAV-mediated gene augmentation therapy, CRISPR technology remains unexplored for this condition 197. Unlike conventional gene replacement approaches, CRISPR enables precise genomic level modification of disease-causing genes. For instance, CRISPR/Cas9-mediated HITI technology holds promise for precisely repairing CSNB-related mutations, potentially achieving lifelong therapeutic benefits from a single treatment.

## Conclusion and Outlook

This review provides an in-depth exploration of the innovative applications of CRISPR technology in the treatment of ocular diseases. The precise gene-editing capabilities of CRISPR have demonstrated significant potential across a range of major ocular conditions, from those that affect the anterior segment to those that affect the posterior segment. This technology not only aids in elucidating pathological mechanisms but also promotes the advancement of personalized therapies. However, the clinical application of CRISPR is not without challenges, particularly concerning off-target effects and ethical considerations, which necessitate further safety validation and regulatory measures. Nevertheless, CRISPR is poised to lead the future of precision medicine in ophthalmology, resulting in revolutionary changes in the field.

The application of CRISPR technology in ophthalmic diseases undoubtedly marks a new chapter in precision medicine. Precision medicine emphasizes tailoring treatment plans based on the unique genomic characteristics of individuals, enabling the formulation of specific therapeutic strategies aligned with patients' genetic profiles. CRISPR not only facilitates the editing of known pathogenic genes but can also be combined with genomic sequencing technologies to enable a comprehensive analysis of a patient's genomic data, thereby identifying potential pathogenic mutations. Additionally, with the rapid development of artificial intelligence (AI), breakthroughs in CRISPR applications within ophthalmology are expected to accelerate further. AI can assist in the design and optimization of gRNAs, significantly enhancing the editing efficiency and specificity of CRISPR systems. AI and machine learning techniques can be employed to identify new gene targets, predict the outcomes of gene editing, and monitor potential off-target effects, thereby improving the comprehensive application of CRISPR gene editing.

Notably, as the clinical applications of CRISPR have expanded in ophthalmology, it is imperative to consider the ethical and legal challenges that accompany these advancements to ensure the safety and affordability of the technology. Establishing robust international standards and regulatory frameworks is crucial to mitigate potential ethical disputes and protect patient rights. Only under a stringent ethical framework and legal guidance can CRISPR technology be safely and responsibly applied in clinical practice, ultimately providing tangible health benefits to a wide range of patients.

The rapid advancement of scientific technology is remarkable, with concepts such as silicon-based life, brain-machine interfaces, and AI robots becoming a reality. CRISPR technology has the potential to revolutionize the field of ophthalmology, transitioning from science fiction to reality. It promises to bring about multifaceted, innovative changes that transcend current treatment limitations and create a new medical paradigm.

## Figures and Tables

**Table 1:** Applications of CRISPR/Cas systems in ocular diseases.

Disease	Target	Executor	Vector	Delivery	Model	Strategy	Key findings	Ref
**Anterior eye disease**								
**Corneal scar and corneal wound healing**	*Sox2*	dCas9	Nake Plasmid	Intracameral injection	Sprague-dawley rats	Gene activation	Activating *SOX2* using CRISPR/Cas9 technology can achieve functional regeneration of the corneal endothelium.	75
	*Aqp5*	Cas9	Not mentioned	Not mentioned	AQP5 knockout (AQP5^-/-^) mice	Knockout	AQP5 promotes corneal epithelial wound healing and nerve regeneration by activating the NGF/Akt signaling pathway.	76
	*Tgif1*	Cas9/ dCas9	Not mentioned	Chemical Transfection	Human corneal stromal fibroblasts	Knockout and activation	CRISPR-mediated editing of the *TGIF1* gene activation inhibits the conversion of corneal fibroblasts to myofibroblasts.	77
**HSK**	*ICP0*	SpCas9	Lentiviral particles	lentivirus transduction	L7 cell	Knockout	CRISPR/Cas9 targets and cleaves the *ICP0* gene, suppressing ICP0 expression to inhibit viral replication and protect cells from HSV-1 infection.	85
	*ICP0 & ICP4*	SpCas9/SaCas9	AAV1	Adeno-associated virus transduction	Primary TG neuronal	Knockout	AAV1-mediated SaCas9 gene editing can effectively inhibit HSV-1 replication in trigeminal ganglion neurons by targeting the *ICP4* gene.	86
	*UL8 &UL29*	SpCas9	HELP	Intrastromal injection	HSV-1 infected mouse HSK model	Knockout	HELP effectively suppresses HSV-1 replication in mouse corneas and trigeminal ganglia by targeting the *UL8* and *UL29* genes.	20
	*NECTIN-1*	Cas9	Lentiviral particles	Not mentioned	HCECs	knockdown	After knocking down *NECTIN-1* in HCECs *via* the CRISPR/Cas9 system, the HSV-1 DNA content dropped to 30% of the control group's level.	88
**Corneal dystrophy**	T*GFBI*	Cas9	Plasmid	Transfection	Human corneal keratocytes derived from a GCD2 patient	HDR	CRISPR/Cas9-mediated HDR successfully corrected the *TGFBI* R124H mutation.	94
	*Col8a2*	SpCas9	Adenovirus	Anterior chamber injection	Col8a2 mutant mice	knockdown	A single intraocular injection of Ad-Cas9-Col8a2gRNA efficiently prevented endothelial cell loss, rescued corneal endothelium pumping function in adult Col8a2 mutant mice.	96
**CoNV**	*Vegfa*	SpCas9	AAV2/9	Subconjunctival injection	Suture-induced mouse CoNV model	Knockout	AAV2/9-mediated CRISPR/Cas9 knockout of the *VEGFA* gene significantly inhibits pathologic CoNV.	99
**Midsection eye disease**								
**Glaucoma**	*MYOC*	Cas9	Adenovirus	Intravitreal injection	Tg-*MYOC*^Y437H^ mice	Knockout	Using CRISPR/Cas9 to disrupt the mutant *MYOC* gene reduces endoplasmic reticulum stress, lowers intraocular pressure, and prevents further glaucomatous damage in mouse eyes.	46
	*Car2*	SpCas9	AAV ShH10	Intravitreal injection	Glaucoma mice model	Knockout	Using CRISPR/Cas9 to disrupt the *Car2* gene shows promise for achieving the goal of “one-shot treatment, lifelong benefits” for glaucoma.	105
**Congenital aniridia**	*Pax6*	SpCas9	Not mentioned	Cotransfection	Human limbal epithelial cells	Knock-in	The study successfully modeled aniridia-related keratopathy *in vitro* using CRISPR/Cas9 gene editing.	108
	*Pax6*	SpCas9	Not mentioned	Electroporation/microinjection	Sey mouse model/*F*ey mouse model	Germline correction	Germline CRISPR/Cas9-mediated gene editing prevents vision loss in a *F*ey mouse model of Aniridia	109
	*Pax6*	ABE8e	LNP	Not mentioned	Fey embryonic mouse cortical neurons	Base editing	ABE8e restored 24.8% of Pax6 expression in *ex vivo* neurons, laying the foundation for translating CRISPR therapy to human aniridia patients.	110
**Posterior eye disease**								
**LCA**	*CEP290*	SpCas9	AAV5	Subretinal injection	Wild-type mice	Knockout	AAV5-CRISPR/Cas9 system can delete the homologous *CEP290* intronic fragment in wild-type mice retinas *via* subretinal injection. Also, a self-limiting CRISPR/Cas9 system is developed to restrict the sustained expression of SpCas9.	115
	*CEP290*	SaCas9	AAV5	Subretinal injection	humanized *CEP290* mice	Knockout	Subretinal delivery of EDIT-101 in humanized *CEP290* mice showed rapid and sustained* CEP290* gene editing.	170
	*Kcnj13*	ABE8e	Silica nanocapsules	Subretinal injection	LCA16 mouse model	Base editing	Using Silica nanocapsules to deliver the ABE8e can efficiently correct the W53X mutation in *KCNJ13*, preserve vision in the LCA16 mouse model, and restore Kir7.1 channel function in RPE cells.	118
	*Rpe65*	SpCas9	AAV9	Subretinal injection	Rd12 mice model	HDR	Dual AAV9-delivered CRISPR/Cas9 can achieve HDR-mediated correction and pathogenic mutation deletion in the rd12 mice model.	121
	*Rpe65*	ABE	Lentiviral particles	Subretinal injection	Rd12 mice model	Base editing	ABE corrects the *RPE65* mutation in rd12 mice *via* subretinal injection, restoring its function and visual function to near-normal levels with minimal off-target and indel mutations.	123
**RP**	*Rho*	SaCas9	AAV2/8	Subretinal injection	*RHO* humanized mouse model	Knockout	AAV2/8-CRISPR/SaCas9 system specifically target and knockout the T17M mutant allele of the *RHO* gene, significantly improving retinal function and protecting photoreceptors in *RHO* humanized mice.	132
	*Rho*	SaCas9	AAV2/8	Subretinal injection	P23H rat model	Knockout	AAV2/8-delivered SaCas9/gRNA selectively ablates the *RHO*-P23H mutant allele, preserves long-term vision in P23H rat.	133
	*Rho*	SpCas9	Nake Plasmid	Subretinal injection	P23H mutant mouse model	Knockout	CRISPR/Cas9 achieves efficiently edit the mutant *RHO* gene in P23H mouse retinas.	134
	*Rho*	SpCas9	AAV2/8	Subretinal injection	Humanized h*RHO*^C110R^/h*RHO*^WT^ mice model	Ablation and replacement	CRISPR-based ablation and replacement strategy *via* dual AAV2/8 in humanized adRP mice efficiently knocks out mutant *RHO* and introduces wild-type genes.	137
	*Rho*	SaCas9	AAV9	Subretinal injection	*Rho*-associated adRP mouse model	Reduction and replacement	CRISPR/SaCas9-mediated reduction and replacement strategy effectively restores retinal electrophysiological function in adRP mouse model.	138
	*Rho*	dCas9	AAV5	Subretinal injection	Transgenic Pro23His mutant pigs	CRISPRi	The AAV5-delivered CRISPRi system targets the *RHO* promoter, reducing mutant RHO protein expression, decreasing endoplasmic reticulum stress and apoptosis markers, and preserving retinal function.	139
	*Prpf31*	SpCas9	AAV2-7m8	Subretinal injection	hiPSC-derived RPE cells and retinal organoids	Gene augmentation	*PRPF31* mutations lead to structural and functional defects in hiPSC-derived RPE and photoreceptor cells, while AAV-mediated *PRPF31* gene augmentation restores cellular morphology, improves electrophysiological responses.	141
	*PHD2*	SpCas9	AAV8	Subretinal injection	RP mice model	Knockout	CRISPR editing of *PHD2* can enhance retinal aerobic glycolysis, reduce mitochondrial oxidation, maintain photoreceptor survival and function in both autosomal recessive and dominant RP mouse models.	142
**BCD**	*Cyp4v2*	SaCas9	rAAV2/8	Subretinal injection	Humanized *Cyp4v3* mutant mice	HITI	The CRISPR/SaCas9-HITI system delivered by rAAV2/8 achieves precise integration of *CYP4V2* in h*Cyp4v3*mut/mut mice, improves retinal morphology and electrophysiological responses.	50
**STGD1**	*Abca4*	ABE	AAV9	Subretinal injection	Female cynomolgus macaques	Base editing	An ABE strategy achieves high-level *ABCA4* gene correction in nonhuman primates, with mean editing rates of 75% in cone photoreceptors and 87% in RPE cells.	147
**Retinal neovascularization**	*Vegfa*	Cas13bt3	AAV2-7m8	Intravitreal injection	Mouse model of proliferative retinopathy	RNA silencing	AAV2-7m8 delivered CRISPR/Cas13bt3 efficiently silences VEGFA mRNA, reducing retinal neovascular leakage and vessel density.	155
	*Vegfr2*	SpCas9	AAV2/8	Intravitreal injection	OIR mouse model	Knockout	AAV2/8-mediated CRISPR/SpCas9 editing of the *VEGFR2* gene effectively suppressed pathological retinal angiogenesis in OIR mouse model.	157
	*Vegfr2*	PE	dual non-integrating lentivirus	Intravitreal injection	OIR mouse model	Prime editing	The PE6x system efficiently edits the *VEGFR2* gene, generating DN-VEGFR2 to inhibit pathological retinal angiogenesis.	158
**CNV**	*Vegfa/Hif1α*	LbCpf1	AAV9	Intravitreal injection	Laser-induced CNV mice model	Knockout	AAV9-delivered LbCpf1 targeting *Vegfa* or *Hif1a* significantly reducing the area of laser-induced CNV.	161
	*Vegfa/Hif1α/Vegfr2*	Nme2Cas9	AAV8	Subretinal injection	Laser-induced CNV mouse model	Knockout	AAV8-delivered Nme2Cas9 efficiently edits target genes, with early intervention targeting *Vegfa* significantly reducing CNV area by 49.5%.	162
	*Vegfa*	SpCas9	Lentivirus	Subretinal injection	Laser-induced CNV mouse model	Knockout	The RPE-specific CRISPR/pVMD2-Cas9 system efficiently knockout *Vegfa*, leading to significant regression of laser-induced CNV in mice.	163
**RB**	*Rb1/* *Rbl1*	SpCas9	Not mentioned	Microinjection	*Xenopus tropicalis*	Knockout	CRISPR/Cas9-mediated knockout of *rb1* and *rbl1* in *Xenopus tropicalis* tadpoles leads to rapid and highly penetrant RB development, recapitulating human histopathological features, thus providing an efficient preclinical model.	167
	*Rb1*	SpCas9	Not mentioned	Electroporation	Teratoma model mimicking TRb neural tumors	Knockout	CRISPR/Cas9-mediated knockout of *RB1* leads to mitochondrial dysfunction, with teratomas recapitulating TRb-like neural expansion, providing a novel model for TRb.	169

**Abbreviations:** CRISPR: clustered regularly interspaced short palindromic repeats; Cas: CRISPR associated; NGF: nerve growth factor; TG: trigeminus; AAV: adeno-associated virus; HELP: HSV-1-erasing lentiviral particles; HSK: herpetic stromal keratitis; HSV: herpes simplex virus; HDR: homology-directed repair; hCECs: human corneal endothelial cells; GCD2: granular corneal dystrophy2; Ad: adenovirus; CoNV: corneal neovascularization; VEGF: vascular endothelial growth factor; ABE: adenine base editor; PE: prime editor; LCA: leber congenital amaurosis; RP: retinitis pigmentosa; CRISPRi: CRISPR interference; BCD: bietti crystalline corneoretinal dystrophy; HITI: homology independent targeted integration; RPE: retinal pigment epithelium; CNV: choroidal neovascularization; DN-VEGFR2: dominant-negative VEGF receptor 2; RB: retinoblastoma; TRb: trilateral retinoblastoma; STGD1: stargardt disease.

**Table 2:** The advantages and disadvantages of CRISPR delivery systems and their applications in ocular diseases.

Category	Applications	Cargo options	Advantages	Limitations
**Physical**				
Microinjection	Congenital aniridia 109/RB 167	DNA/mRNA/ proteins	High-precision single-cell delivery/Low off-target risk/Suitability for hard-to-transfect cells.	Labor-intensive operation with substantial damage/Low throughput/Stage-dependency
Electroporation	Corneal scar and corneal wound healing 75/ Congenital aniridia 108/ LCA 119/RP 134/STGD1 149/Retinal neovascularization 157	DNA/mRNA/ proteins	Efficient batch processing/Low mechanical damage/Enhanced HDR efficiency	Potential cytotoxicity/Slightly higher off-target risk
**Viral Delivery**				
AAVs	HSK 86/CoNV 99/ Glaucoma 105/ LCA 115/RP 133/ BCD 50/STGD1 147/ Retinal neovascularization 155/CNV 161	DNA	High biosafety/Low immunogenicity/Tissue tropism/Stable episomal expression/Clinical validation	Small packaging capacity/Preexisting antibody restriction/Potential integration risk
AVs	Corneal dystrophy 96/ Glaucoma 46	DNA	Large packaging capacity/Efficient transduction/Episomal expression/Scalable production	High immunogenicity/Short expression duration
LVs	LCA 123/ RP 132/Retinal neovascularization 158 /CNV 163	RNA	Large-capacity integration/Efficient *in vitro* screening	Random integration risk/Long-term off-target effects/ High immunogenicity
**Non-Viral Delivery**				
CPPs	Not mentioned	DNA/mRNA/ Proteins	Rapid action/High stability/Low cytotoxicity	Prone to proteolytic degradation/Endosomal escape barrier/Insufficient targeting
LNPs	Congenital aniridia 110	DNA/mRNA/ proteins	Efficient endosomal escape/Compatible with multiple delivery forms/Low immunogenicity	Targeting depends on ligands/Particle size limitation/Difficult production standardization
Polymers	Not mentioned	RNPs	Flexible modification/High loading capacity/Biocompatibility	Cytotoxicity/Poor serum stability/Low nuclear transport efficiency
DNA nanoclew	Not mentioned	RNPs	Sequence programmability/Precise design/Stimuli-responsive release	Exhibits certain immunogenicity/Poor stability/Low *in vivo* delivery efficiency
AuNPs	Not mentioned	RNPs	Strong nucleic acid binding and protection/Cell penetrability/Low immunogenicity	Potential cytotoxicity/Insufficient targeting
VLPs	HSK 20	RNPs/RNA	No integration risk/Transient expression safety/High loading capacity	*In vivo* stability challenge/Tissue penetration limitation

**Abbreviations:** AAV: adeno-associated virus; AVs: adenovirus; LVs: lentivirus; CPPs: cell-penetrating peptide; LNPs: lipid nanoparticles; AuNPs: gold nanoparticles; VLPs: virus-like particles; CoNV: corneal neovascularization; HSK: herpetic stromal keratitis; LCA: leber congenital amaurosis; RP: retinitis pigmentosa; CNV: choroidal neovascularization; RB: retinoblastoma; STGD1: stargardt disease.

## Abbreviations

Cas: CRISPR associated
gRNA: guide RNA
RP: retinitis pigmentosa
LCA: leber congenital amaurosis
HSV: herpes simplex virus
CoNV: corneal neovascularization
VEGF: vascular endothelial growth factor
CRISPR: clustered regularly interspaced short palindromic repeats
crRNA: CRISPR RNA
PAM: protospacer adjacent motif
NHEJ: non-homologous end joining
HDR: homology-directed repair
ABE: adenine base editor
CBE: cytosine base editor
PE: prime editor
hCECs: human corneal endothelial cells
SOX2: sex-determining region Y-box 2
AQP5: aquaporin 5
NGF: nerve growth factor
hCSFs: human corneal stromal fibroblasts
CMFs: corneal myofibroblasts
HK: herpetic keratitis
HSK: herpetic stromal keratitis
HELP: HSV-1 erasing lentiviral particles
mLPs: mRNA-carrying lentiviral particles
NECTIN-1: nectin cell adhesion molecule 1
TBCD: thiel-behnke corneal dystrophy
GCD: granular corneal dystrophy
FECD: fuchs' endothelial corneal dystrophy
hUVECs: human umbilical vein endothelial cells
RGCs: retinal ganglion cells
TM: trabecular meshwork
LSCs: limbal stem cells
LNPs: lipid nanoparticles
RPE: retinal pigment epithelium
hiPSCs: human induced pluripotent stem cells
Rho: rhodopsin
CRISPRi: CRISPR interference
PHD: prolyl hydroxylase
VHL: von hippel-lindau
HIF: hypoxia-inducible factor
hfCas13X: high-fidelity Cas13X
BCD: bietti crystalline corneoretinal dystrophy
HITI: homology independent targeted integration
VECs: vascular endothelial cells
DN-VEGFR2: dominant-negative VEGF receptor 2
NILVs: non-integrative lentiviral vectors
RB: retinoblastoma
TRb: trilateral retinoblastoma
STGD1: stargardt disease
AAVs: adeno-associated virus
AVs: adenovirus
LVs: lentivirus
VLPs: virus-like particles
FDA: Food and Drug Administration
VEP: visual evoked potentials
AI: artificial intelligence
